# Efficacy of post‐first‐line agents for advanced gastrointestinal stromal tumors following imatinib failure: A network meta‐analysis

**DOI:** 10.1002/cam4.5912

**Published:** 2023-04-21

**Authors:** Kehan Hu, Hu Zhang, Mingrong Shu, Xingyue Wang

**Affiliations:** ^1^ Department of Gastroenterology, West China Hospital Sichuan University Chengdu China; ^2^ Centre for Inflammatory Bowel Disease, Institution of Inflammation and Immunity, West China Hospital Sichuan University Chengdu China; ^3^ Lab of Inflammatory Bowel Disease, Frontiers Science Center for Disease‐Related Molecular Network, West China Hospital Sichuan University Chengdu China; ^4^ Department of Infection Control, West China Hospital Sichuan University Chengdu China; ^5^ Department of Graduate Medical Education, West China School of Medicine Sichuan University Chengdu China

**Keywords:** clinical efficacy, gastrointestinal stromal tumor, imatinib failure, network meta‐analysis, post‐first‐line therapy

## Abstract

**Background:**

Imatinib is the standard first‐line treatment for advanced gastrointestinal stromal tumors (GISTs); however, most patients eventually develop imatinib resistance, leading to considerable clinical challenges. Few direct comparisons have been made between different post‐first‐line therapies on clinical efficacy in advanced GIST following imatinib failure.

**Methods:**

Databases including PubMed, Embase, Scopus, Google Scholars, and Cochrane Library from inception to February 2023 were retrieved for randomized controlled trials evaluating the clinical efficacy of different post‐first‐line agents for advanced GIST following imatinib failure. Network and conventional meta‐analysis were carried out using Stata/MP 16.0.

**Results:**

Ripretinib showed significant improvement in progression‐free survival (PFS) rates from the 2nd to the 12th month compared to placebo, while there was virtually no evidence that the rest active agents had a significant benefit at the 12th month. Masitinib, ripretinib, sunitinib, regorafenib, and pimitespib exhibited significantly longer median PFS than placebo, and pairwise comparisons indicated there were no significant differences among masitinib, ripretinib, and sunitinib. These post‐first‐line agents decreased the risk of disease progression or death by 65% (HR = 0.35, 95% CI: 0.26–0.47) compared to placebo. Ripretinib and sunitinib came into effect earlier and exhibited more consistent overall survival (OS) rate improvements than masitinib and pimitespib, while pairwise comparisons revealed no significant differences in these four active agents concerning the improvement in OS rate. These post‐first‐line agents decreased the risk of death by 39% (HR = 0.61, 95% CI: 0.44–0.83) over placebo for advanced GIST following imatinib failure.

**Conclusion:**

The active agents in our analysis as post‐first‐line therapies are able to provide superior clinical efficacy, with improved PFS rate and OS rate at certain time points, as well as absolute values of PFS and OS for advanced GIST. Ripretinib might be the optimal recommendation as a post‐first‐line treatment for advanced GIST following imatinib failure.

## Graphical abstract

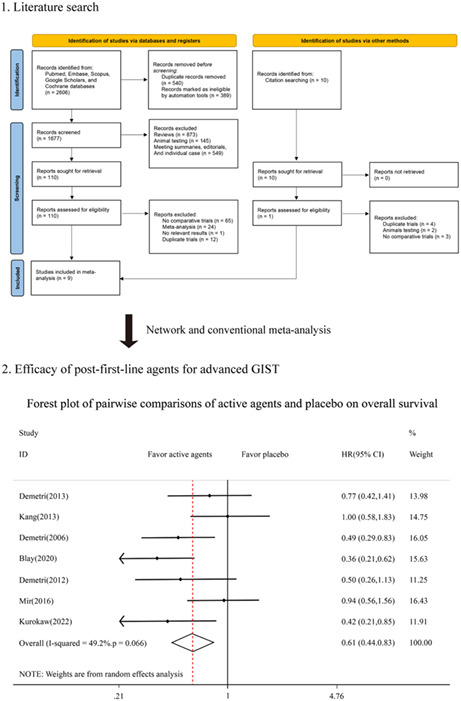

## 1 INTRODUCTION

Gastrointestinal stromal tumors (GISTs) are the most common mesenchymal tumors of the digestive tract, with an incidence of 1.0–1.5/100,000 individuals worldwide; they predominantly involve the stomach (50%–60%) and the small intestine (30%–35%). Most patients diagnosed with GIST are in their 60s, and the gender distribution is fairly equal.^1^, ^2^, ^3^, ^4^

GIST patients are usually asymptomatic or have nonspecific symptoms, including abdominal distension, abdominal pain, early satiety, bleeding, fatigue, and palpable mass, which may lead to delay in diagnosis and treatment.^5^ The spread in GIST is usually expansive and mainly occurs through hematogenous routes.^6^ GIST is associated with a poor prognosis and is insensitive to radiation and cytotoxic chemotherapy.^7^, ^8^ The standard treatment for GIST is surgery, but recurrences do occur.^9^ For advanced GIST (metastatic, unresectable, or recurrent), there is no curative therapy, and the emergence of targeted molecular therapy has revolutionized treatment. In recent years, remarkable achievements have been made in the development of tyrosine kinase inhibitors (TKIs) that target KIT and/or platelet‐derived growth factor receptor α (PDGFRA); this has led to major clinical improvements and improved survival among advanced GIST patients.^10^, ^11^, ^12^, ^13^, ^14^, ^15^

Imatinib, a multitargeted TKI targeting KIT and PDGFR, has been widely used as the standard first‐line treatment for advanced GIST, improving the median survival from 1‐to 5 years. However, most patients inevitably develop imatinib resistance often after 20–24 months on imatinib, leading to disease progression and eventually death, and since the approval of imatinib in 2002, there have been no improvements in first‐line therapy for GIST; this has resulted in significant challenges in the clinical management of advanced GIST.^16^, ^17^, ^18^, ^19^, ^20^, ^21^, ^22^, ^23^ For advanced GIST patients for whom imatinib therapy has been unsuccessful, sunitinib, and regorafenib are the current second‐and third‐line therapies, respectively.^24^ Unfortunately, almost all advanced GIST patients with imatinib resistance ultimately develop sunitinib resistance, usually within 1 year.^20^, ^25^ The US Food and Drug Administration (FDA) authorized ripretinib as a fourth‐line treatment for advanced GIST in May 2020.^26^ Pimitespib, an oral heat shock protein 90 inhibitor, has shown its efficacy on advanced GIST with significantly improved progression‐free survival (PFS) following imatinib failure as a post‐first‐line therapy.^27^

There is an unmet medical need for better therapeutic alternatives which can improve the current condition of advanced GIST patients, and to provide more options and clinical benefits for such patients following first‐line imatinib failure.

Although an increasing number of studies concerning post‐first‐line treatments for advanced GIST have been conducted, few direct comparisons have been made between these active agents in terms of their treatment efficacy, and no definite conclusions have been drawn concerning their effectiveness. Considering this, we conducted this meta‐analysis incorporating both conventional meta‐analysis and network meta‐analysis, with the aim of summarizing and comparing the clinical efficacy of post‐first‐line therapeutic agents in terms of PFS rate and OS rate at certain time points, as well as absolute values of PFS and OS for advanced GIST after first‐line imatinib therapy failure.

## 2 METHODS

### 2.1 Search strategy

We initiated our study by searching the PubMed, Embase, Scopus, Google Scholars, and Cochrane Library databases for randomized controlled trials (RCTs) from their inception to February 2023, using the following keywords: “gastrointestinal stromal tumor” or “GIST”; “advanced” or “metastatic” or “unresectable” or “recurrent,” and “imatinib”. Furthermore, we explored the references of relevant reviews and guidelines for additional candidate publications. Two reviewers screened titles and abstracts independently to verify all potentially eligible publications for full‐text assessment. The final studies included were identified on the basis of the inclusion and exclusion criteria described above. This network meta‐analysis followed the guidelines of PRISMA.

### 2.2 Inclusion criteria

The eligible studies were verified according to the following criteria by two experienced investigators individually: (1) the study design must be RCT; (2) patients were diagnosed with advanced GIST and previously experienced first‐line imatinib therapy failure (caused by either disease progression or intolerance); (3) patients received an active agent as post‐first‐line treatment in the research group and an active agent as post‐first‐line treatment or placebo in the control group; (4) the clinical efficacy outcomes were PFS rate and OS rate at certain time points, as well as absolute values of PFS and OS, and atleast one desirable outcome must be reported.

### 2.3 Exclusion criteria

Articles were excluded if they were reviews, single‐arm studies, case reports, animal studies, commentaries, conference abstracts, retrospective observational studies, or studies published in a language other than English.

### 2.4 Data extraction and analysis

Information extracted included first author, year of publication, study design, comparative treatment regimens, sample size, age, and sex. In addition, HRs and 95% CIs for PFS and OS were extracted. We also extracted the PFS rate and OS rate at certain time points from the PFS and OS curves using GetData software. Publication bias was assessed by using Egger's test, and *p* < 0.05 indicated the potential of publication bias. We utilized a network funnel plot to evaluate possible small sample effects in a network meta‐analysis. For each RCT, two investigators independently extracted the data and evaluated the risk of bias according to the Cochrane Collaboration's risk of bias tool.^28^

### 2.5 Statistical analysis

For dichotomous variables, we expressed effects as odds ratios (ORs), while for continuous variables, effects were expressed as weighted mean differences (WMDs) with their 95% CIs. Risks were presented as HRs with 95% CIs. Continuous variables, when presented as the median with the interquartile range, were converted to the mean.^29^ Both a fixed‐effects model and a random‐effects model were adopted in this meta‐analysis. The surface under the cumulative ranking curve (SUCRA) was applied to rank regimens based on their probabilities of being the optimal choice for each outcome. The higher the SUCRA value, the higher the possibility of greater efficacy.^30^ A matrix was generated in order to verify whether the difference between every two agents with corresponding SUCRA values was statistically significant. To enhance the stability of each result, overall and loop consistency and inconsistency were examined. All statistical analyses were conducted using Stata 16.0/MP, and *p* < 0.05 was considered statistically significant.

## 3 RESULTS

### 3.1 Literature search and risk of bias assessment

**FIGURE 1:**
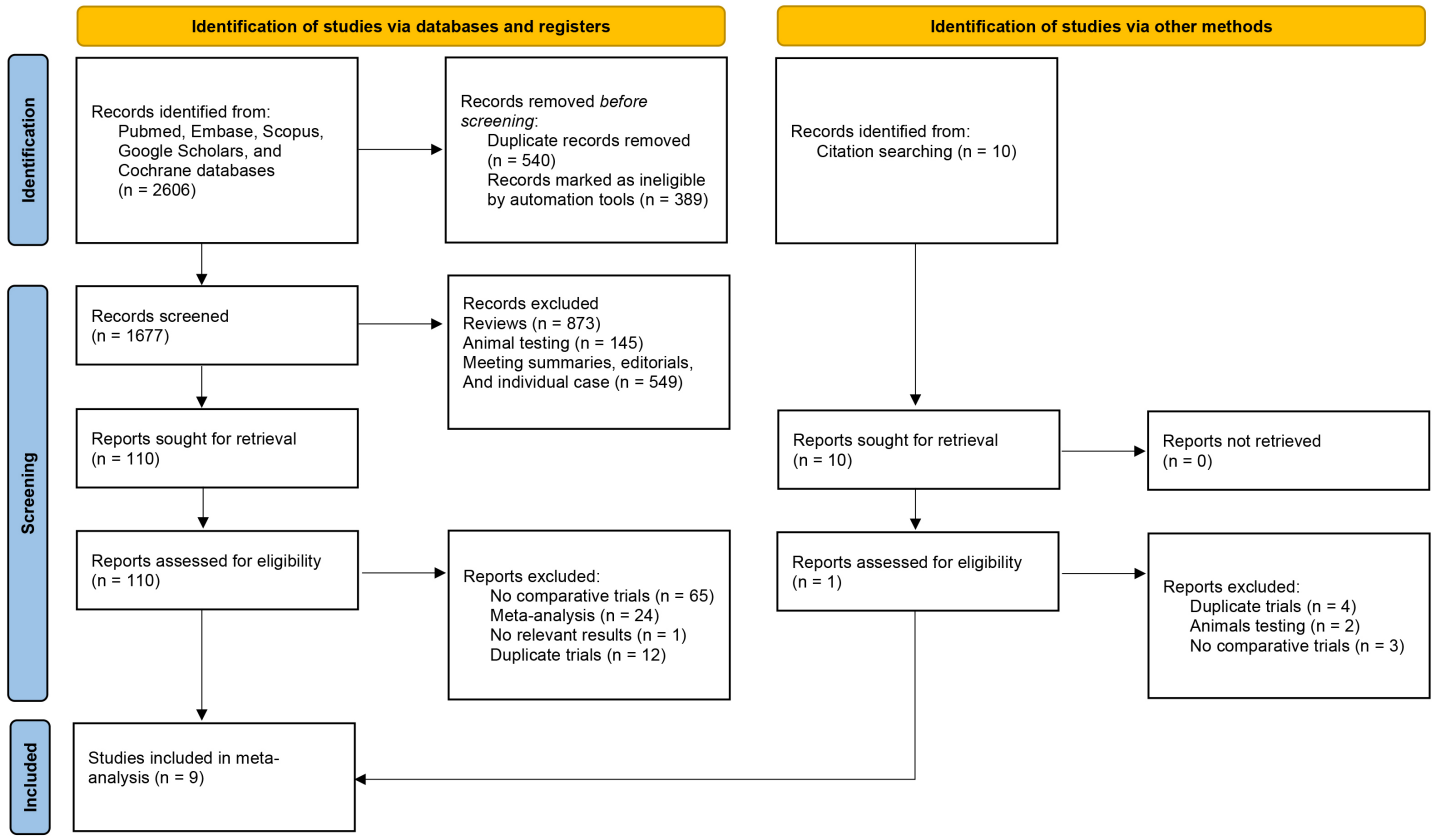
Flow diagram of study search and selection for the meta‐analysis.

The flow diagram presents the details of the literature screening and selection process for the network meta‐analysis (Figure 1). Our search strategy involved the retrieval of a total of 2606 publications, of which nine studies^15^, ^26^, ^27^, ^31^, ^32^, ^33^, ^34^, ^35^, ^36^ were finally included in the meta‐analysis after removal of duplicates and screening of titles, abstracts, and full‐text, involving 1746 patients with advanced GIST. Table 1 provides an overview of the characteristics of these studies. Details of the risk of bias graph and the risk of bias summary are reported in Figures S1 and S2, respectively.

**TABLE 1:** Characteristics of included studies for the meta‐analysis.

First author	Year of publication	Study design	Regimens being compared	Sample size	Age, median years (range)	Sex (male, %)	PFS HR (95% CI)	OS HR (95% CI)
Demetri^31^	2013	RCT	Regorafenib	133	60.0 (18.0–82.0)	63.9	0.27	0.77
			Placebo	66	61.0 (25.0–87.0)	63.6	(0.19–0.39)	(0.42–1.41)
Kang^32^	2013	RCT	Imatinib	41	57.0 (52.0–65.0)	71.0	0.46	1.00
			Placebo	40	61.0 (54.0–67.0)	65.0	(0.27–0.78)	(0.58–1.83)
Demetri^33^	2006	RCT	Sunitinib	207	58.0 (23.0–84.0)	63.8	0.33	0.49
			Placebo	105	55.0 (23.0–81.0)	61.0	(0.23–0.47)	(0.29–0.83)
Blay^26^	2020	RCT	Ripretinib	85	59.0 (29.0–82.0)	55.0	0·15	0.36
			Placebo	44	65.0 (33.0–83.0)	59.0	(0·09–0·25)	(0.21–0.62)
Adenis^34^	2014	RCT	Masitinib	23	62.0 (31.0–82.0)	48.0	1.10	0.27
			Sunitinib	21	67.0 (41.0–85.0)	52.0	(0.60–2.20)	(0.09–0.85)
Demetri^35^	2012	RCT	Sunitinib	243	57.0 (23.0–84.0)	63.0	0.347	0.505
			Placebo	118	55.0 (23.0–81.0)	60.0	(0.253–0.475)	(0.262–1.134)
Mir^36^	2016	RCT	Pazopanib	40	65.0 (33.0–85.0)	63.0	0.59	0.94
			Placebo	41	59.0 (27.0–81.0)	78.0	(0.37–0.96)	(0.56–1.56)
Bauer^15^	2022	RCT	Ripretinib	226	59.5 (18.0–86.0)	61.5	1.05	NA
			Sunitinib	227	60.0 (26.0–88.0)	62.6	(0.82–1.33)	NA
Kurokaw^27^	2022	RCT	Pimitespib	58	62.0 (55.0–77.0)	58.6	0.51	0.42
			Placebo	28	61.5 (51.0–68.5)	53.4	(0.30–0.87)	(0.21–0.85)

Abbreviations: CI, confidence interval; HR, hazard ratio; NA, not available; OS, overall survival; PFS, progression‐free survival; RCT, randomized controlled trial.

### 3.2 Progression‐free survival rate

**FIGURE 2:**
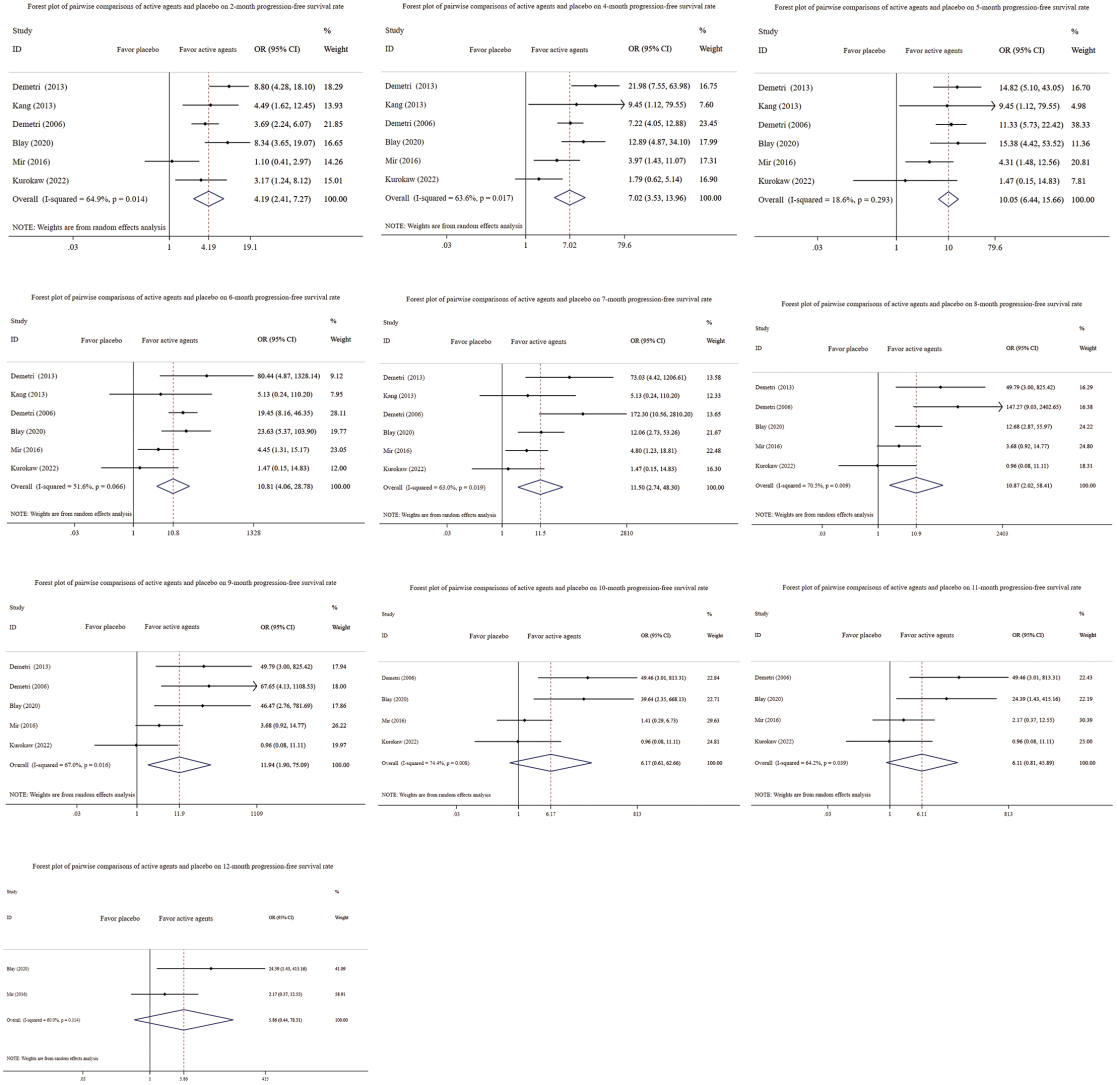
Forest plot of pairwise comparisons of active agents and placebo on progression‐free survival rate at certain time points.

PFS rates at certain time points have not been extensively explored by previous studies. The present study included a comparison of PFS rate results at certain time points separately; the results are shown in Figure 2. Using a conventional meta‐analysis, we analyzed a total of six studies^26^, ^27^, ^31^, ^32^, ^33^, ^36^ involving 888 patients and including six different active agents and placebo.

In the meta‐analysis, significant benefit of active agents compared with placebo was confirmed in 2 month (OR = 4.19, 95% CI: 2.41–7.27), 4 month (OR = 7.02, 95% CI: 3.53–13.96), 5 month (OR = 10.05, 95% CI: 6.44–15.66), 6 month (OR = 10.81, 95% CI: 4.06–28.78), 7 month (OR = 11.50, 95% CI: 2.74–48.30), 8 month (OR = 10.87, 95% CI: 2.02–58.41), and 9 month (OR = 11.94, 95% CI: 1.90–75.09). However, there was no significant improvement in 10 month (OR = 6.17, 95% CI: 0.61–62.66), 11 month (OR = 6.11, 95% CI: 0.81–45.89), and 12 month PFS rate (OR = 5.86, 95% CI: 0.44–78.31). The most effective treatment in terms of PFS rate was found to be ripretinib. In comparison with the placebo, this treatment showed a significant improvement in PFS rate from the 2nd to the 12th month. Moreover, there was virtually no evidence that the rest active agents had significant effect at the 12th month compared with the placebo (Figure 2).

### 3.3 Progression‐free survival

In total, we analyzed nine studies^15^, ^26^, ^27^, ^31^, ^32^, ^33^, ^34^, ^35^, ^36^ involving 1746 patients including eight different regimens using a network meta‐analysis on the median PFS. The network graph is shown in Figure S3, which shows sunitinib compared with the placebo was the most comparison. The matrix suggested that masitinib (WMD = 6.07, 95% CI: 4.53–7.61), ripretinib (WMD = 4.93, 95% CI: 3.97–5.89), sunitinib (WMD = 4.86, 95% CI: 4.08–5.64), regorafenib (WMD = 3.92, 95% CI: 2.68–5.15), and pimitespib (WMD = 1.41, 95% CI: 0.17–2.65) all showed median PFS improvements compared to the placebo; while pazopanib (WMD = 1.10, 95% CI: −0.16–2.37) and imatinib (WMD = 0.91, 95% CI: −0.33–2.15) did not show significant improvements. In addition, pairwise comparisons indicated that masitinib, ripretinib, sunitinib, and regorafenib all showed significant improvements in PFS when compared to pimitespib, and masitinib performed better than regorafenib in the clinical efficacy of PFS; while the median PFS did not differ significantly among patients treated with masitinib, ripretinib, and sunitinib (Table 2).

**TABLE 2:** Matrix of each pairwise comparison of all the agents on progression‐free survival (shown as WMD and 95% CI).

	Masitinib	Ripretinib	Sunitinib	Regorafenib	Pimitespib	Pazopanib	Imatinib	Placebo
SUCRA (%)	98.1	79.1	76.9	60.2	33.4	27.3	23.1	2.0
Masitinib	0	−1.13 (−2.77, 0.50)	−1.21 (−2.53, 0.12)	−2.15 (−4.12, −0.18)	−4.66 (−6.63, −2.68)	−4.96 (−6.96, −2.97)	−5.16 (−7.14, −3.18)	−6.07 (−7.61, −4.53)
Ripretinib	1.13 (−0.50, 2.77)	0	−0.07 (−1.03, 0.89)	−1.02 (−2.58, 0.55)	−3.52 (−5.09, −1.95)	−3.83 (−5.42, −2.24)	−4.02 (−5.60, −2.45)	−4.93 (−5.89, −3.97)
Sunitinib	1.21 (−0.12, 2.53)	0.07 (−0.89, 1.03)	0	−0.95 (−2.41, 0.52)	−3.45 (−4.92, −1.98)	−3.76 (−5.24, −2.27)	−3.95 (−5.42, −2.48)	−4.86 (−5.64, −4.08)
Regorafenib	2.15 (0.18, 4.12)	1.02 (−0.55, 2.58)	0.95 (−0.52, 2.41)	0	−2.50 (−4.25, −0.75)	−2.81 (−4.58, −1.05)	−3.01 (−4.76, −1.25)	−3.92 (−5.15, −2.68)
Pimitespib	4.66 (2.68, 6.63)	3.52 (1.95, 5.09)	3.45 (1.98, 4.92)	2.50 (0.75, 4.25)	0	−0.31 (−2.08, 1.46)	−0.50 (−2.26, 1.25)	−1.41 (−2.65, −0.17)
Pazopanib	4.96 (2.97, 6.96)	3.83 (2.24, 5.42)	3.76 (2.27, 5.24)	2.81 (1.05, 4.58)	0.31 (−1.46, 2.08)	0	−0.19 (−1.97, 1.58)	−1.10 (−2.37, 0.16)
Imatinib	5.16 (3.18, 7.14)	4.02 (2.45, 5.60)	3.95 (2.48, 5.42)	3.01 (1.25, 4.76)	0.50 (−1.25, 2.26)	0.19 (−1.58, 1.97)	0	−0.91 (−2.15, 0.33)
Placebo	6.07 (4.53, 7.61)	4.93 (3.97, 5.89)	4.86 (4.08, 5.64)	3.92 (2.68, 5.15)	1.41 (0.17, 2.65)	1.10 (−0.16, 2.37)	0.91 (−0.33, 2.15)	0

*Note*: Yellow indicated a relative treatment benefit, blue indicated a relative treatment harm, pink indicated no significant.

Abbreviations: CI, confidence interval; SUCRA, surface under the cumulative ranking curve; WMD, weighted mean difference.

We analyzed a total of seven studies^26^, ^27^, ^31^, ^32^, ^33^, ^35^, ^36^ involving 1249 patients and included six different active agents and placebo using a conventional meta‐analysis on HR of PFS. The results obtained with a random‐effects model showed that these active agents as a post‐first‐line therapy significantly decreased the risk of disease progression or death by 65% in comparison to the placebo (HR = 0.35, 95% CI: 0.26–0.47) for advanced GIST patients following imatinib therapy failure (Figure S4).

### 3.4 Overall survival rate

In total, we analyzed eight^26^, ^27^, ^31^, ^32^, ^33^, ^34^, ^35^, ^36^ studies involving 1293 patients and included eight different regimens using a network meta‐analysis on OS rates at certain time points. The network graph is shown in Figure S5; and sunitinib compared with the placebo was the most common comparison. Table 3 summarizes the OS rate improvements at certain time points for seven different active agents when compared with the placebo, and the box marked in yellow and pink indicated that there was significant improvement and no significant improvement between the active agent and the placebo in terms of OS rate efficacy according to the OR with 95% CI, respectively. Ripretinib and sunitinib showed an improved OS rate from the 2nd month to the 10th month, while masitinib and pimitespib exhibited OS rate improvements from the 4th month to the 10th month and from the 6th month to the 9th month, respectively. Masitinib and pimitespib were slower to achieve a significant improvement in OS rate, and the efficacy of masitinib seemed to be unsteady due to interruption in the improvement of OS rate at the 8th month. In comparison to masitinib, the OS rate improvements for ripretinib and sunitinib not only occurred earlier but also persisted longer, while for all these three active agents, the improvements in OS rate declined around the 11th month. Furthermore, pazopanib, regorafenib, and imatinib were unable to provide OS rate improvements in our analysis compared to the placebo (Table 3).

**TABLE 3:** The efficacy of active agents on overall survival rate at certain time points summary.

Month	Agent						
	Masitinib	Pazopanib	Pimitespib	Regorafenib	Ripretinib	Sunitinib	Imatinib
2‐month							
4‐month							
5‐month							
6‐month							
7‐month							
8‐month							
9‐month							
10‐month							
11‐month							

*Note*: The box marked in yellow and pink indicated that there was significant improvement and no significant improvement between the active agent and placebo on overall survival rate efficacy according to the odds ratio with 95% CI, respectively.

The matrix summarizes the pairwise comparison of OS rates at certain time points for masitinib, pazopanib, pimitespib, placebo, regorafenib, ripretinib, sunitinib, and imatinib. The matrix revealed the following results for the various active agents in comparison to the placebo at different time points: ripretinib (OR = 5.21, 95% CI: 1.50–18.04) and sunitinib (OR = 2.78, 95% CI: 1.44–5.37) show 2 month OS rate improvements; masitinib (OR = 11.57, 95% CI: 1.07–125.57), ripretinib (OR = 4.98, 95% CI: 1.91–12.98) and sunitinib (OR = 3.16, 95% CI: 2.06–4.86) show 4 month OS rate improvements; masitinib (OR = 15.59, 95% CI: 1.54–157.67), ripretinib (OR = 5.11, 95% CI: 2.14–12.20) and sunitinib (OR = 3.02, 95% CI: 2.03–4.50) show 5 month OS rate improvements; masitinib (OR = 18.08, 95% CI: 1.87–175.23), ripretinib (OR = 4.62, 95% CI: 2.00–10.66), pimitespib (OR = 4.04, 95% CI: 1.40–11.72) and sunitinib (OR = 2.64, 95% CI: 1.81–3.86) show 6 month OS rate improvements; masitinib (OR = 9.00, 95% CI: 1.36–59.68), pimitespib (OR = 3.60, 95% CI: 1.18–11.00), ripretinib (OR = 3.34, 95% CI: 1.35–8.24) and sunitinib (OR = 2.75, 95% CI: 1.67–4.52) show 7 month OS rate improvements; pimitespib (OR = 4.42, 95% CI: 1.27–15.46), ripretinib (OR = 3.77, 95% CI: 1.26–11.28) and sunitinib (OR = 2.14, 95% CI: 1.11–4.13) show 8 month OS rate improvements; masitinib (OR = 5.65, 95% CI: 1.13–28.29), ripretinib (OR = 3.16, 95% CI: 1.48–6.73), sunitinib (OR = 2.72, 95% CI: 1.93–3.82), and pimitespib (OR = 2.56, 95% CI: 1.01–6.49) show 9 month OS rate improvements; and masitinib (OR = 7.60, 95% CI: 1.82–31.78), ripretinib (OR = 3.81, 95% CI: 1.77–8.20) and sunitinib (OR = 2.60, 95% CI: 1.87–3.63) show 10 month OS rate improvements (Tables S1–S9).

Overall, masitinib, pimitespib, ripretinib, and sunitinib were observed to provide significantly improved OS rates at certain time points compared with the placebo, while the pairwise comparisons revealed there to be no statistically significant differences among these treatment groups in terms of the OS rate improvement (Tables S1–S9).

### 3.5 Overall survival

**FIGURE 3:**
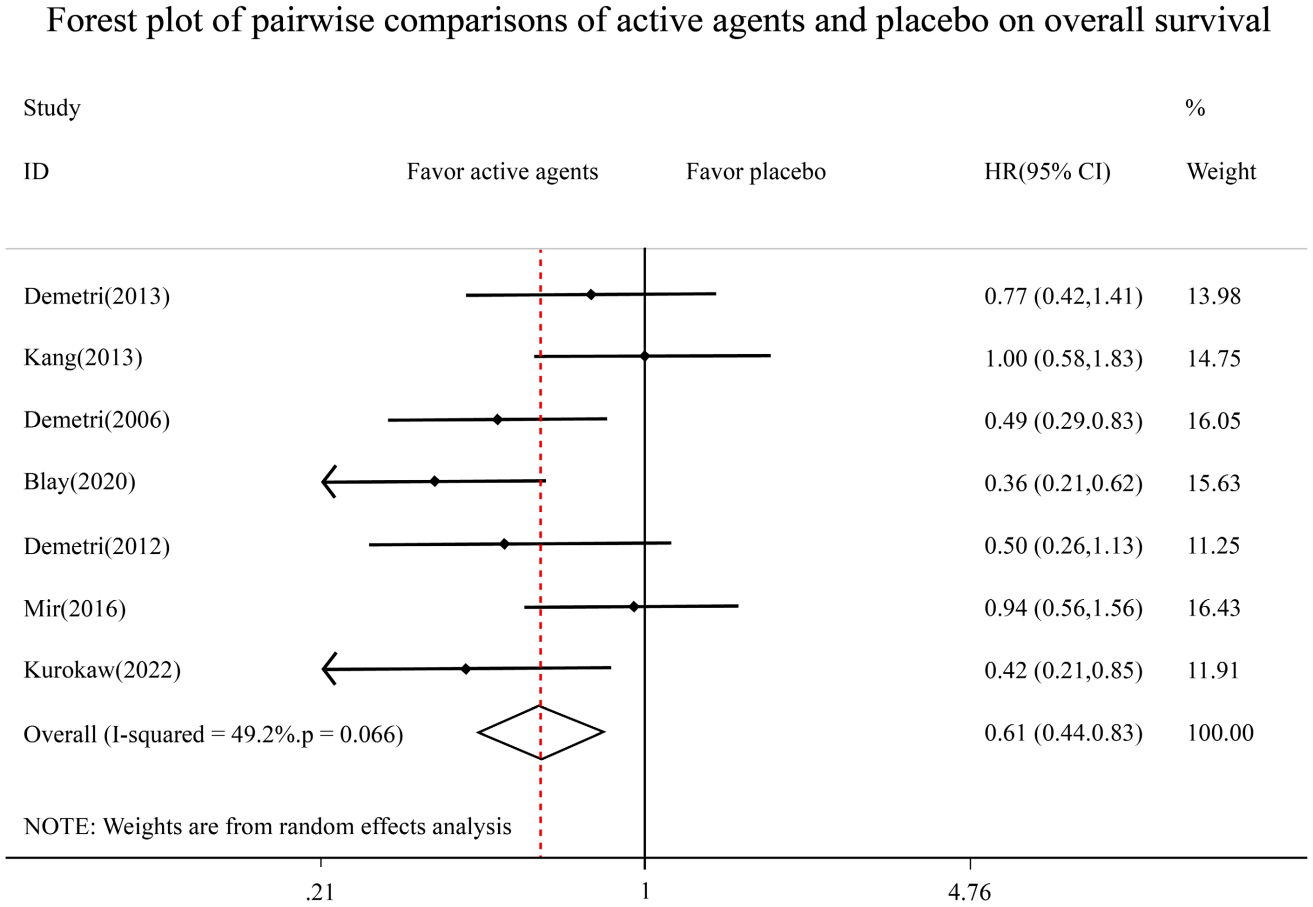
Forest plot of pairwise comparisons of active agents and placebo on overall survival.

We analyzed a total of seven^26^, ^27^, ^31^, ^32^, ^33^, ^35^, ^36^ studies involving 1249 patients and included six different active agents and a placebo using a conventional meta‐analysis on HR of OS. The results obtained using a random‐effects model showed that these active agents significantly reduced the risk of death by 39% in comparison to the placebo (HR = 0.61, 95% CI: 0.44–0.83) for advanced GIST patients following first‐line imatinib therapy failure (Figure 3).

### 3.6 Publication bias and small sample effect

No publication bias was identified in the PFS rate (*p* > 0.05) using Egger's test, and there was also no significant publication bias in the HRs of PFS and OS (PFS: *p* = 0.687; OS: *p* = 0.611, respectively). Additionally, the network funnel plot of pairwise comparisons on PFS and OS rate at certain time points are summarized in Figures S6 and S7, respectively. The scattered points distributed at the bottom of the network funnel plot indicated that there were small sample effects (Figures S6 and S7).

## 4 DISCUSSION

To the best of our knowledge, this is the first network meta‐analysis to assess the clinical efficacy of post‐first‐line agents for the treatment of advanced GIST following first‐line imatinib therapy failure comprehensively and precisely, involving PFS rate and OS rate at certain time points as well as absolute values of PFS and OS.

Indeed, the most striking finding is that ripretinib and sunitinib came into effect earlier and showed a more persistent improvement in OS rate compared to masitinib and pimitespib. Ripretinib, as an FDA‐approved fourth‐line agent for advanced GIST, the efficacy on OS rate and median PFS was comparable to that of second‐line sunitinib therapy.^24^, ^26^ Additionally, ripretinib was found to be the most effective treatment in terms of PFS rate as it showed a significant improvement in PFS rate from the 2nd to the 12th month, while there was virtually no evidence that sunitinib had a significant benefit at the 12th month. Finally, the active agents in our analysis as post‐first‐line treatments revealed a significant decrease in the risk of disease progression or death, with an HR of 35% in comparison to the placebo. Meanwhile, a 39% reduction in risk of death was observed with these active agents for advanced GIST patients following imatinib treatment failure.

As indicated above, ripretinib might be the optimal recommendation as a post‐first‐line treatment for advanced GIST following imatinib therapy failure in our analysis. However, our results are somewhat inconsistent with those of another study.^37^ One explanation for these inconsistent results could be that several studies involved in the above meta‐analysis did not meet prespecified inclusion criteria. Another explanation could be that we focused on different metrics and used different statistical methods.

In GIST, the most common primary mutations occur on exons 9 or 11 of the KIT gene.^38^ Imatinib, an inhibitor targeting exons 9/11‐mutated KIT gene, is the first‐line therapy for advanced GIST, while most imatinib‐treated patients ultimately relapse, possibly due to the outgrowth of clones carrying KIT mutations associated with imatinib resistance, typically involve KIT exons 13/14 (ATP‐binding pocket) and 17/18 (activation loop).^39^, ^40^, ^41^ Notably, the potency of ripretinib against KIT mutations observed in imatinib‐resistant patients was found to be superior to that of sunitinib in vitro, suggesting that ripretinib may be premium in post‐first‐line GIST. Ripretinib, a switch‐control TKI, broadly inhibits wild‐type KIT/PDGFA mutations and multiple primary and secondary KIT/PDGFRA mutations associated with drug‐resistant GIST. On the other hand, sunitinib is active against mutations in KIT exons 13/14 but not exons 17/18,^15^, ^19^, ^26^, ^42^, ^43^ which may explain the findings in our analysis that ripretinib might be the optimal recommendation as a post‐first‐line treatment for advanced GIST following imatinib failure. Furthermore, ripretinib was recommended as a second‐line treatment for advanced GIST following first‐line imatinib failure in the CSCO diagnosis and treatment guidelines for GISTs (2021).^44^ Thus, ripretinib has emerged as a promising choice of treatment for advanced GIST.

The results from the INTRIGUE study comparing ripretinib with sunitinib on efficacy and safety as a second‐line treatment option for patients with advanced GIST following imatinib treatment has been reported recently. In this study, ripretinib did not show superiority over sunitinib in terms of PFS, but showed meaningful clinical activity for imatinib‐treated advanced GIST patients.^15^ The objective response rate and median PFS for the ripretinib group were 21.7% and 8 months, respectively, while for the sunitinib group they were 17.6% and 8.3 months, respectively; however, no significant difference was observed. Ripretinib (41.3%) induced fewer Grade 3–4 treatment‐emergent adverse events than sunitinib (65.6%), including hypertension and palmar‐plantar erythrodysesthesia syndrome. The superior safety of ripretinib means that occurrences of adverse events are reduced and compliance for patients is improved, ensuring continuous and adequate drug therapy. Nevertheless, repretinib had a more favorable safety profile. Additionally, ripretinib displayed better tolerability than sunitinib, providing meaningful clinical benefit.^43^, ^45^, ^46^

Ripretinib was a clinically meaningful, well‐tolerated, and favorable safety profile strategy for advanced GIST patients following first‐line imatinib failure. Additionally, the efficacy of ripretinib in terms of OS rate and PFS was comparable to and the safety was better than that of sunitinib.^15^ For advanced GIST patients, when first‐line imatinib therapy failure, physicians may choose ripretinib other than sunitinib as post‐first line treatment.

This network meta‐analysis contained several potential limitations. First, the number of publications eligible for inclusion in this meta‐analysis was relatively small, while several studies involved relatively small sample sizes, which could potentially affect the precision of the analysis. Second, there were some shortcomings in the indirect comparative analysis used to evaluate the clinical curative effect of the different agents in our analysis; thus, further direct comparative trials are needed. Third, there was a degree of heterogeneity among the studies, possibly arising from age, sex, ethnicity, and follow‐up duration. Based on the above limitation, further large‐scale head‐to‐head RCTs should be conducted to confirm the generalizability of the findings of the present study.

## 5 CONCLUSION

This network meta‐analysis aimed to evaluate and compare the clinical efficacy of several active agents as a post‐first‐line therapeutic option for advanced GIST patients following first‐line imatinib failure. Our results revealed these active agents to be effective as post‐first‐line treatments in improving the clinical efficacy outcomes of PFS rate and OS rate at certain time points as well as absolute values of PFS and OS. Ripretinib may be the optimal recommendation for advanced GIST patients following imatinib failure. Given the limitations described above, in order to further verify these findings, large‐scale head‐to‐head RCTs should be conducted to confirm those findings.

## AUTHOR CONTRIBUTIONS

**Kehan Hu:** Conceptualization (equal); data curation (equal); formal analysis (equal); writing – original draft (lead); writing – review and editing (equal). **HU ZHANG:** Data curation (equal); writing – original draft (supporting); writing – review and editing (equal). **Mingrong Shu:** Data curation (equal); formal analysis (equal); writing – review and editing (equal). **xingyue wang:** Conceptualization (equal); data curation (equal); supervision (lead); writing – original draft (supporting); writing – review and editing (equal).

## CONFLICT OF INTEREST STATEMENT

The authors disclose no conflicts of interest associated with this work.

## ETHICS STATEMENT

No ethical approval was required for the current meta‐analysis.

## Supporting information


**Supporting information S1.** Supplementary material

## Data Availability

The data that support the findings of this study are available from the corresponding author upon reasonable request.

## References

[1] Soreide K , Sandvik OM , Soreide JA , et al. Global epidemiology of gastrointestinal stromal tumours (GIST): a systematic review of population‐based cohort studies. Cancer Epidemiol. 2016;40:39‐46. 26618334 10.1016/j.canep.2015.10.031

[2] Joensuu H , Hohenberger P , Corless CL . Gastrointestinal stromal tumour. Lancet. 2013;382(9896):973‐983. 23623056 10.1016/S0140-6736(13)60106-3

[3] Kelly CM , Gutierrez Sainz L , Chi P . The management of metastatic GIST: current standard and investigational therapeutics. J Hematol Oncol. 2021;14(1):2. 33402214 10.1186/s13045-020-01026-6 PMC7786896

[4] Xu H , Zhou S , Hu Q , Cao D . Apatinib treatment for unresectable gastrointestinal stromal tumor with synchronous gastric cancer. Precis Clin Med. 2020;3(1):67‐70. 35693429 10.1093/pcmedi/pbaa005 PMC8985806

[5] Wang MX , Devine C , Segaran N , Ganeshan D . Current update on molecular cytogenetics, diagnosis and management of gastrointestinal stromal tumors. World J Gastroenterol. 2021;27(41):7125‐7133. 34887632 10.3748/wjg.v27.i41.7125 PMC8613640

[6] Kim JJ , Lim JY , Nguyen SQ . Laparoscopic resection of gastrointestinal stromal tumors: does laparoscopic surgery provide an adequate oncologic resection? World J Gastrointest Endosc. 2017;9(9):448‐455. 28979709 10.4253/wjge.v9.i9.448 PMC5605344

[7] Glod J , Arnaldez FI , Wiener L , et al. A phase II trial of vandetanib in children and adults with succinate dehydrogenase‐deficient gastrointestinal stromal tumor. Clin Cancer Res. 2019;25(21):6302‐6308. 31439578 10.1158/1078-0432.CCR-19-0986 PMC6825553

[8] Zalcberg JR . Ripretinib for the treatment of advanced gastrointestinal stromal tumor. Therap Adv Gastroenterol. 2021;14:17562848211008177. 10.1177/17562848211008177 PMC8053826 33948116

[9] Brinch CM , Aggerholm‐Pedersen N , Hogdall E , Krarup‐Hansen A . Medical oncological treatment for patients with gastrointestinal stromal tumor (GIST)–a systematic review. Crit Rev Oncol Hematol. 2022;172:103650. 35283299 10.1016/j.critrevonc.2022.103650

[10] Corless CL , Barnett CM , Heinrich MC . Gastrointestinal stromal tumours: origin and molecular oncology. Nat Rev Cancer. 2011;11(12):865‐878. 22089421 10.1038/nrc3143

[11] Blay JY , Kang YK , Nishida T , von Mehren M . Gastrointestinal stromal tumours. Nat Rev Dis Primers. 2021;7(1):22. 33737510 10.1038/s41572-021-00254-5

[12] Mantese G . Gastrointestinal stromal tumor: epidemiology, diagnosis, and treatment. Curr Opin Gastroenterol. 2019;35(6):555‐559. 31577561 10.1097/MOG.0000000000000584

[13] Casali PG , Abecassis N , Aro HT , et al. Gastrointestinal stromal tumours: ESMO‐EURACAN clinical practice guidelines for diagnosis, treatment and follow‐up. Ann Oncol. 2018;29(Suppl 4):iv68‐iv78. 29846513 10.1093/annonc/mdy095

[14] Weissman SM . Precision medicine: a few thoughts from 2022. Precision Clin Med. 2022;5(1):pbac003. 10.1093/pcmedi/pbac003 PMC9036555 35692446

[15] Bauer S , Jones RL , Blay JY , et al. Ripretinib versus sunitinib in patients with advanced gastrointestinal stromal tumor after treatment with imatinib (INTRIGUE): a randomized, open‐label, phase III trial. J Clin Oncol. 2022;40(34):3918‐3928. 35947817 10.1200/JCO.22.00294 PMC9746771

[16] Koo DH , Ryu MH , Kim KM , et al. Asian consensus guidelines for the diagnosis and management of gastrointestinal stromal tumor. Cancer Res Treat. 2016;48(4):1155‐1166. 27384163 10.4143/crt.2016.187 PMC5080813

[17] Bauer S , Joensuu H . Emerging agents for the treatment of advanced, imatinib‐resistant gastrointestinal stromal tumors: current status and future directions. Drugs. 2015;75(12):1323‐1334. 26187774 10.1007/s40265-015-0440-8 PMC4532715

[18] Debiec‐Rychter M , Cools J , Dumez H , et al. Mechanisms of resistance to imatinib mesylate in gastrointestinal stromal tumors and activity of the PKC412 inhibitor against imatinib‐resistant mutants. Gastroenterology. 2005;128(2):270‐279. 15685537 10.1053/j.gastro.2004.11.020

[19] Nemunaitis J , Bauer S , Blay JY , et al. Intrigue: Phase III study of ripretinib versus sunitinib in advanced gastrointestinal stromal tumor after imatinib. Future Oncol. 2020;16(1):4251‐4264. 31755321 10.2217/fon-2019-0633

[20] Ran L , Sirota I , Cao Z , et al. Combined inhibition of MAP kinase and KIT signaling synergistically destabilizes ETV1 and suppresses GIST tumor growth. Cancer Discov. 2015;5(3):304‐315. 25572173 10.1158/2159-8290.CD-14-0985 PMC4355391

[21] Vitiello GA , Bowler TG , Liu M , et al. Differential immune profiles distinguish the mutational subtypes of gastrointestinal stromal tumor. J Clin Invest. 2019;129(5):1863‐1877. 30762585 10.1172/JCI124108 PMC6486334

[22] Seifert AM , Zeng S , Zhang JQ , et al. PD‐1/PD‐L1 blockade enhances T‐cell activity and antitumor efficacy of imatinib in gastrointestinal stromal tumors. Clin Cancer Res. 2017;23(2):454‐465. 27470968 10.1158/1078-0432.CCR-16-1163 PMC5241182

[23] Serrano C , García‐Del‐Muro X , Valverde C , et al. Clinicopathological and molecular characterization of metastatic gastrointestinal stromal tumors with prolonged benefit to frontline imatinib. Oncologist. 2019;24(5):680‐687. 30126859 10.1634/theoncologist.2018-0032 PMC6516132

[24] Zhang JQ , Zeng S , Vitiello GA , et al. Macrophages and CD8(+) T cells mediate the antitumor efficacy of combined CD40 ligation and imatinib therapy in gastrointestinal stromal tumors. Cancer Immunol Res. 2018;6(4):434‐447. 29467128 10.1158/2326-6066.CIR-17-0345 PMC6203303

[25] Rovithi M , Gerritse SL , Honeywell RJ , et al. Phase I dose‐escalation study of once weekly or once every two weeks administration of high‐dose sunitinib in patients with refractory solid tumors. J Clin Oncol. 2019;37(5):411‐418. 30586316 10.1200/JCO.18.00725 PMC6368417

[26] Blay J‐Y , Serrano C , Heinrich MC , et al. Ripretinib in patients with advanced gastrointestinal stromal tumours (INVICTUS): a double‐blind, randomised, placebo‐controlled, phase 3 trial. Lancet Oncol. 2020;21(7):923‐934. 32511981 10.1016/S1470-2045(20)30168-6 PMC8383051

[27] Kurokawa Y , Honma Y , Sawaki A , et al. Pimitespib in patients with advanced gastrointestinal stromal tumor (CHAPTER‐GIST‐301): a randomized, double‐blind, placebo‐controlled phase III trial. Ann Oncol. 2022;33(9):959‐967. 35688358 10.1016/j.annonc.2022.05.518

[28] Higgins JP , Altman DG , Gøtzsche PC , et al. The Cochrane Collaboration's tool for assessing risk of bias in randomised trials. BMJ. 2011;343:d5928. 22008217 10.1136/bmj.d5928 PMC3196245

[29] Musso G , Cassader M , Paschetta E , Gambino R . Thiazolidinediones and advanced liver fibrosis in nonalcoholic steatohepatitis: a meta‐analysis. JAMA Intern Med. 2017;177(5):633‐640. 28241279 10.1001/jamainternmed.2016.9607 PMC5470366

[30] Ricci C , Pagano N , Taffurelli G , et al. Comparison of efficacy and safety of 4 combinations of laparoscopic and intraoperative techniques for management of gallstone disease with biliary duct calculi: a systematic review and network meta‐analysis. JAMA Surg. 2018;153(7):e181167. 29847616 10.1001/jamasurg.2018.1167 PMC6137518

[31] Demetri GD , Reichardt P , Kang Y‐K , et al. Efficacy and safety of regorafenib for advanced gastrointestinal stromal tumours after failure of imatinib and sunitinib (GRID): an international, multicentre, randomised, placebo‐controlled, phase 3 trial. Lancet. 2013;381(9863):295‐302. 23177515 10.1016/S0140-6736(12)61857-1 PMC3819942

[32] Kang Y‐K , Ryu M‐H , Yoo C , et al. Resumption of imatinib to control metastatic or unresectable gastrointestinal stromal tumours after failure of imatinib and sunitinib (RIGHT): a randomised, placebo‐controlled, phase 3 trial. Lancet Oncol. 2013;14(12):1175‐1182. 24140183 10.1016/S1470-2045(13)70453-4 PMC4347867

[33] Demetri GD , van Oosterom AT , Garrett CR , et al. Efficacy and safety of sunitinib in patients with advanced gastrointestinal stromal tumour after failure of imatinib: a randomised controlled trial. The Lancet. 2006;368(9544):1329‐1338. 10.1016/S0140-6736(06)69446-4 17046465

[34] Adenis A , Blay JY , Bui‐Nguyen B , et al. Masitinib in advanced gastrointestinal stromal tumor (GIST) after failure of imatinib: a randomized controlled open‐label trial. Ann Oncol. 2014;25(9):1762‐1769. 25122671 10.1093/annonc/mdu237 PMC4143095

[35] Demetri GD , Garrett CR , Schoffski P , et al. Complete longitudinal analyses of the randomized, placebo‐controlled, phase III trial of sunitinib in patients with gastrointestinal stromal tumor following imatinib failure. Clin Cancer Res. 2012;18(11):3170‐3179. 22661587 10.1158/1078-0432.CCR-11-3005 PMC4030710

[36] Mir O , Cropet C , Toulmonde M , et al. Pazopanib plus best supportive care versus best supportive care alone in advanced gastrointestinal stromal tumours resistant to imatinib and sunitinib (PAZOGIST): a randomised, multicentre, open‐label phase 2 trial. Lancet Oncol. 2016;17(5):632‐641. 27068858 10.1016/S1470-2045(16)00075-9

[37] Zhang X , Liang Y , Li Y , Yin J . Comparative efficacy and safety of different regimens of advanced gastrointestinal stromal tumors after failure prior tyrosine kinase inhibitors: a network meta‐analysis. Adv Ther. 2021;38(1):399‐412. 33131035 10.1007/s12325-020-01545-1

[38] Janku F , Bauer S , Shoumariyeh K , et al. Efficacy and safety of ripretinib in patients with KIT‐altered metastatic melanoma. ESMO Open. 2022;7(4):100520. 35753087 10.1016/j.esmoop.2022.100520 PMC9434165

[39] Wagner AJ , Severson PL , Shields AF , et al. Association of combination of conformation‐specific KIT inhibitors with clinical benefit in patients with refractory gastrointestinal stromal tumors: a phase 1b/2a nonrandomized clinical trial. JAMA Oncol. 2021;7(9):1343‐1350. 34236401 10.1001/jamaoncol.2021.2086 PMC8267845

[40] Yuzawa S , Opatowsky Y , Zhang Z , Mandiyan V , Lax I , Schlessinger J . Structural basis for activation of the receptor tyrosine kinase KIT by stem cell factor. Cell. 2007;130(2):323‐334. 17662946 10.1016/j.cell.2007.05.055

[41] Demetri GD , von Mehren M , Blanke CD , et al. Efficacy and safety of imatinib mesylate in advanced gastrointestinal stromal tumors. N Engl J Med. 2002;347(7):472‐480. 12181401 10.1056/NEJMoa020461

[42] Smith BD , Kaufman MD , Lu WP , et al. Ripretinib (DCC‐2618) is a switch control kinase inhibitor of a broad spectrum of oncogenic and drug‐resistant KIT and PDGFRA variants. Cancer Cell. 2019;35(5):738‐751 e9. 31085175 10.1016/j.ccell.2019.04.006

[43] Poh A . Testing ripretinib against sunitinib in GIST. Cancer Discov. 2022;12(3):591‐592. 35086925 10.1158/2159-8290.CD-NB2022-0004

[44] Guidelines working Committee of Chinese Society of Clinical Oncology . Chinese Society of Clinical Oncology (CSCO) diagnosis and treatment guidelines for gastrointestinal stromal tumors (2021). People's Health Publishing House; 2021.

[45] Gold JS , Gönen M , Gutiérrez A , et al. Development and validation of a prognostic nomogram for recurrence‐free survival after complete surgical resection of localised primary gastrointestinal stromal tumour: a retrospective analysis. Lancet Oncol. 2009;10(11):1045‐1052. 19793678 10.1016/S1470-2045(09)70242-6 PMC3175638

[46] Heinrich MC , Jones RL , Gelderblom H , et al. INTRIGUE: a phase III, randomized, open‐label study to evaluate the efficacy and safety of ripretinib versus sunitinib in patients with advanced gastrointestinal stromal tumor previously treated with imatinib. Presented at: ASCO Plenary Series: January 26, 2022 Session. 2022.

