# Supplementary material for: Efficacy of post‐first‐line agents for advanced gastrointestinal stromal tumors following imatinib failure: A network meta‐analysis
Source: Cancer Med. 2023 Apr 21;12(11):12187–97. doi: 10.1002/cam4.5912 (PMC10278495; doi:10.1002/cam4.5912)
Supplement: Supplementary file 1 — Supporting information S1. Supplementary material [file CAM4-12-12187-s001.docx]

**Supplementary materials**

**1. Table S1.** Matrix of each pairwise comparison of all the agents on 2-month overall survival rate (shown as OR and 95% CI).

**2.** **Table S2**. Matrix of each pairwise comparison of all the agents on 4-month overall survival rate (shown as OR and 95% CI).

**3. Table S3.** Matrix of each pairwise comparison of all the agents on 5-month overall survival rate (shown as OR and 95% CI).

**4. Table S4.** Matrix of each pairwise comparison of all the agents on 6-month overall survival rate (shown as OR and 95% CI).

**5. Table S5.** Matrix of each pairwise comparison of all the agents on 7-month overall survival rate (shown as OR and 95% CI).

**6. Table S6.** Matrix of each pairwise comparison of all the agents on 8-month overall survival rate (shown as OR and 95% CI).

**7. Table S7.** Matrix of each pairwise comparison of all the agents on 9-month overall survival rate (shown as OR and 95% CI).

**8. Table S8.** Matrix of each pairwise comparison of all the agents on 10-month overall survival rate (shown as OR and 95% CI).

**9. Table S9.** Matrix of each pairwise comparison of all the agents on 11-month overall survival rate (shown as OR and 95% CI).

**10. Figure S1.** Risk of bias graph.

**11. Figure S2.** Risk of bias summary; “+” (green) and “-” (red) represent low and high risk of bias, respectively.

**12. Figure S3.** Network graph of each pairwise comparison on progression-free survival.

**13. Figure S4.** Forest plot of pairwise comparisons of active agents and placebo on progression-free survival.

**14. Figure S5.** Network graph of each pairwise comparison on overall survival rate at certain time points.

**15.** **Figure S6.** Network funnel plot of pairwise comparisons on progression-free survival; A, Masitinib; B, Pazopanib; C, Pimitespib; D, Placebo; E, Regorafenib; F, Ripretinib; G, Sunitinib; H, Imatinib.

**16.** **Figure S7.** Network funnel plot of pairwise comparisons on overall survival rate at certain time points; (a) 2-month, (b) 4-month, (c) 5-month, (d) 6-month, (e) 7-month, (f) 8-month, (g) 9-month, (h) 10-month, (i) 11-month; A, Masitinib; B, Pazopanib; C, Pimitespib; D, Placebo; E, Regorafenib; F, Ripretinib; G, Sunitinib; H, Imatinib.

| Table S1. Matrix of each pairwise comparison of all the agents on 2-month overall survival rate (shown as OR and 95% CI). | | | | | | | | |
| --- | --- | --- | --- | --- | --- | --- | --- | --- |
|  | Ripretinib | Sunitinib | Pimitespib | Regorafenib | Imatinib | Placebo | Masitinib | Pazopanib |
| SCURA (%) | 89.5 | 75.9 | 58.0 | 52.5 | 39.7 | 36.5 | 24.3 | 23.6 |
| Ripretinib | 1 | 0.53 (0.13,2.18) | 0.39 (0.01,24.67) | 0.28 (0.05,1.58) | 0.20 (0.03,1.34) | 0.19 (0.06,0.67) | 0.10 (0.01,1.35) | 0.09 (0.01,1.41) |
| Sunitinib | 1.88 (0.46,7.66) | 1 | 0.74 (0.01,40.36) | 0.53 (0.14,2.07) | 0.37 (0.07,1.84) | 0.36 (0.19,0.70) | 0.18 (0.02,1.69) | 0.17 (0.01,2.14) |
| Pimitespib | 2.54 (0.04,158.76) | 1.35 (0.02,73.87) | 1 | 0.72 (0.01,44.26) | 0.50 (0.01,33.62) | 0.49 (0.01,25.19) | 0.24 (0.00,23.85) | 0.23 (0.00,23.95) |
| Regorafenib | 3.53 (0.63,19.69) | 1.88 (0.48,7.32) | 1.39 (0.02,85.66) | 1 | 0.70 (0.11,4.57) | 0.68 (0.21,2.22) | 0.34 (0.02,4.65) | 0.32 (0.02,4.86) |
| Imatinib | 5.07 (0.74,34.46) | 2.70 (0.54,13.41) | 2.00 (0.03,134.09) | 1.44 (0.22,9.43) | 1 | 0.97 (0.23,4.19) | 0.49 (0.03,7.63) | 0.46 (0.03,7.94) |
| Placebo | 5.21 (1.50,18.04) | 2.78 (1.44,5.37) | 2.05 (0.04,106.11) | 1.48 (0.45,4.84) | 1.03 (0.24,4.42) | 1 | 0.50 (0.05,5.16) | 0.47 (0.04,5.46) |
| Masitinib | 10.41 (0.74,146.49) | 5.55 (0.59,52.10) | 4.10 (0.04,401.81) | 2.95 (0.22,40.47) | 2.06 (0.13,32.24) | 2.00 (0.19,20.63) | 1 | 0.95 (0.03,27.82) |
| Pazopanib | 10.96 (0.71,169.65) | 5.85 (0.47,73.28) | 4.32 (0.04,447.23) | 3.11 (0.21,46.90) | 2.16 (0.13,37.20) | 2.11 (0.18,24.18) | 1.05 (0.04,30.84) | 1 |

Abbreviations: OR, odds ratio; CI, confidence interval; SCURA, surface under the cumulative ranking curve. Yellow indicated a relative treatment benefit, blue indicated a relative treatment harm, pink indicated no significant.

| Table S2. Matrix of each pairwise comparison of all the agents on 4-month overall survival rate (shown as OR and 95% CI). | | | | | | | | |
| --- | --- | --- | --- | --- | --- | --- | --- | --- |
|  | Masitinib | Ripretinib | Sunitinib | Pimitespib | Regorafenib | Pazopanib | Imatinib | Placebo |
| SCURA (%) | 89.1 | 80.0 | 66.0 | 63.4 | 17.0 | 29.3 | 20.5 | 17.0 |
| Masitinib | 1 | 0.43 (0.03,5.63) | 0.27 (0.03,2.85) | 0.29 (0.01,5.94) | 0.13 (0.01,1.62) | 0.11 (0.01,1.71) | 0.09 (0.01,1.18) | 0.09 (0.01,0.94) |
| Ripretinib | 2.32 (0.18,30.36) | 1 | 0.64 (0.22,1.82) | 0.67 (0.08,5.42) | 0.29 (0.08,1.10) | 0.25 (0.05,1.36) | 0.21 (0.05,0.82) | 0.20 (0.08,0.52) |
| Sunitinib | 3.66 (0.35,38.20) | 1.57 (0.55,4.50) | 1 | 1.06 (0.16,7.10) | 0.46 (0.17,1.26) | 0.40 (0.09,1.70) | 0.33 (0.11,0.95) | 0.32 (0.21,0.49) |
| Pimitespib | 3.44 (0.17,70.42) | 1.48 (0.18,11.90) | 0.94 (0.14,6.29) | 1 | 0.43 (0.06,3.40) | 0.37 (0.04,3.77) | 0.31 (0.04,2.50) | 0.30 (0.05,1.89) |
| Regorafenib | 7.94 (0.62,101.75) | 3.42 (0.91,12.78) | 2.17 (0.80,5.92) | 2.31 (0.29,18.09) | 1 | 0.86 (0.16,4.52) | 0.71 (0.19,2.70) | 0.69 (0.28,1.70) |
| Pazopanib | 9.25 (0.58,146.48) | 3.98 (0.73,21.61) | 2.53 (0.59,10.87) | 2.69 (0.27,27.25) | 1.17 (0.22,6.15) | 1 | 0.83 (0.15,4.54) | 0.80 (0.20,3.22) |
| Imatinib | 11.18 (0.85,147.24) | 4.81 (1.22,18.94) | 3.06 (1.05,8.90) | 3.25 (0.40,26.35) | 1.41 (0.37,5.35) | 1.21 (0.22,6.64) | 1 | 0.97 (0.36,2.57) |
| Placebo | 11.57 (1.07,125.57) | 4.98 (1.91,12.98) | 3.16 (2.06,4.86) | 3.36 (0.53,21.38) | 1.46 (0.59,3.61) | 1.25 (0.31,5.04) | 1.03 (0.39,2.75) | 1 |

Abbreviations: OR, odds ratio; CI, confidence interval; SCURA, surface under the cumulative ranking curve. Yellow indicated a relative treatment benefit, blue indicated a relative treatment harm, pink indicated no significant.

| Table S3. Matrix of each pairwise comparison of all the agents on 5-month overall survival rate (shown as OR and 95% CI). | | | | | | | | |
| --- | --- | --- | --- | --- | --- | --- | --- | --- |
|  | Masitinib | Ripretinib | Sunitinib | Pimitespib | Regorafenib | Imatinib | Pazopanib | Placebo |
| SCURA (%) | 93.7 | 82.3 | 65.5 | 61.5 | 35.5 | 28.4 | 18.5 | 14.6 |
| Masitinib | 1 | 0.33 (0.03,3.88) | 0.19 (0.02,1.89) | 0.19 (0.01,2.51) | 0.10 (0.01,1.13) | 0.08 (0.01,1.01) | 0.06 (0.00,0.85) | 0.06 (0.01,0.65) |
| Ripretinib | 3.05 (0.26,36.12) | 1 | 0.59 (0.23,1.54) | 0.57 (0.13,2.49) | 0.30 (0.09,0.97) | 0.25 (0.07,0.91) | 0.19 (0.04,0.85) | 0.20 (0.08,0.47) |
| Sunitinib | 5.16 (0.53,50.40) | 1.69 (0.65,4.40) | 1 | 0.96 (0.27,3.39) | 0.50 (0.20,1.23) | 0.43 (0.16,1.19) | 0.32 (0.09,1.17) | 0.33 (0.22,0.49) |
| Pimitespib | 5.40 (0.40,73.21) | 1.77 (0.40,7.81) | 1.05 (0.29,3.71) | 1 | 0.52 (0.12,2.23) | 0.45 (0.10,2.07) | 0.34 (0.06,1.87) | 0.35 (0.10,1.15) |
| Regorafenib | 10.28 (0.89,119.18) | 3.37 (1.03,11.04) | 1.99 (0.81,4.90) | 1.91 (0.45,8.10) | 1 | 0.86 (0.25,2.95) | 0.64 (0.15,2.78) | 0.66 (0.29,1.48) |
| Imatinib | 11.98 (0.99,145.32) | 3.93 (1.10,14.09) | 2.32 (0.84,6.42) | 2.22 (0.48,10.18) | 1.17 (0.34,4.01) | 1 | 0.75 (0.16,3.49) | 0.77 (0.30,1.96) |
| Pazopanib | 16.05 (1.17,220.11) | 5.26 (1.17,23.67) | 3.11 (0.86,11.29) | 2.97 (0.53,16.56) | 1.56 (0.36,6.77) | 1.34 (0.29,6.26) | 1 | 1.03 (0.30,3.51) |
| Placebo | 15.59 (1.54,157.67) | 5.11 (2.14,12.20) | 3.02 (2.03,4.50) | 2.89 (0.87,9.62) | 1.52 (0.68,3.40) | 1.30 (0.51,3.32) | 0.97 (0.29,3.31) | 1 |

Abbreviations: OR, odds ratio; CI, confidence interval; SCURA, surface under the cumulative ranking curve. Yellow indicated a relative treatment benefit, blue indicated a relative treatment harm, pink indicated no significant.

| Table S4. Matrix of each pairwise comparison of all the agents on 6-month overall survival rate (shown as OR and 95% CI). | | | | | | | | |
| --- | --- | --- | --- | --- | --- | --- | --- | --- |
|  | Masitinib | Ripretinib | Pimitespib | Sunitinib | Regorafenib | Imatinib | Pazopanib | Placebo |
| SCURA (%) | 95.1 | 79.1 | 73.8 | 60.1 | 37.2 | 23.2 | 16.9 | 14.7 |
| Masitinib | 1 | 0.26 (0.02,2.87) | 0.22 (0.02,2.75) | 0.15 (0.02,1.37) | 0.09 (0.01,0.95) | 0.06 (0.01,0.73) | 0.05 (0.00,0.64) | 0.06 (0.01,0.54) |
| Ripretinib | 3.92 (0.35,44.08) | 1 | 0.88 (0.23,3.39) | 0.57 (0.23,1.43) | 0.34 (0.11,1.04) | 0.25 (0.07,0.84) | 0.21 (0.06,0.78) | 0.22 (0.09,0.50) |
| Pimitespib | 4.47 (0.36,54.91) | 1.14 (0.29,4.42) | 1 | 0.65 (0.21,2.02) | 0.39 (0.11,1.42) | 0.29 (0.07,1.14) | 0.24 (0.06,1.04) | 0.25 (0.09,0.72) |
| Sunitinib | 6.85 (0.73,64.29) | 1.75 (0.70,4.38) | 1.53 (0.50,4.74) | 1 | 0.59 (0.26,1.36) | 0.44 (0.17,1.14) | 0.37 (0.12,1.08) | 0.38 (0.26,0.55) |
| Regorafenib | 11.53 (1.06,125.66) | 2.94 (0.96,9.00) | 2.58 (0.71,9.43) | 1.68 (0.73,3.87) | 1 | 0.74 (0.23,2.33) | 0.62 (0.18,2.16) | 0.64 (0.30,1.34) |
| Imatinib | 15.65 (1.37,178.80) | 4.00 (1.19,13.46) | 3.50 (0.88,13.93) | 2.29 (0.88,5.96) | 1.36 (0.43,4.29) | 1 | 0.84 (0.22,3.20) | 0.87 (0.36,2.09) |
| Pazopanib | 18.68 (1.56,224.36) | 4.77 (1.28,17.71) | 4.18 (0.96,18.12) | 2.73 (0.93,8.02) | 1.62 (0.46,5.67) | 1.19 (0.31,4.56) | 1 | 1.03 (0.38,2.84) |
| Placebo | 18.08 (1.87,175.23) | 4.62 (2.00,10.66) | 4.04 (1.40,11.72) | 2.64 (1.81,3.86) | 1.57 (0.75,3.29) | 1.16 (0.48,2.78) | 0.97 (0.35,2.66) | 1 |

Abbreviations: OR, odds ratio; CI, confidence interval; SCURA, surface under the cumulative ranking curve. Yellow indicated a relative treatment benefit, blue indicated a relative treatment harm, pink indicated no significant.

| Table S5. Matrix of each pairwise comparison of all the agents on 7-month overall survival rate (shown as OR and 95% CI). | | | | | | | | |
| --- | --- | --- | --- | --- | --- | --- | --- | --- |
|  | Masitinib | Pimitespib | Ripretinib | Sunitinib | Regorafenib | imatinib | Pazopanib | Placebo |
| SCURA (%) | 91.5 | 73.3 | 71.7 | 64.5 | 40.2 | 23.5 | 21.3 | 13.9 |
| Masitinib | 1 | 0.40 (0.04,3.60) | 0.37 (0.05,3.02) | 0.31 (0.05,1.89) | 0.18 (0.02,1.46) | 0.13 (0.02,1.09) | 0.12 (0.01,1.05) | 0.11 (0.02,0.74) |
| Pimitespib | 2.50 (0.28,22.49) | 1 | 0.93 (0.22,3.90) | 0.76 (0.22,2.59) | 0.46 (0.11,1.86) | 0.32 (0.07,1.43) | 0.30 (0.06,1.40) | 0.28 (0.09,0.85) |
| Ripretinib | 2.70 (0.33,21.94) | 1.08 (0.26,4.54) | 1 | 0.82 (0.29,2.31) | 0.50 (0.15,1.70) | 0.35 (0.09,1.33) | 0.32 (0.08,1.30) | 0.30 (0.12,0.74) |
| Sunitinib | 3.27 (0.53,20.31) | 1.31 (0.39,4.45) | 1.21 (0.43,3.41) | 1 | 0.61 (0.23,1.60) | 0.42 (0.14,1.28) | 0.39 (0.12,1.27) | 0.36 (0.22,0.60) |
| Regorafenib | 5.41 (0.69,42.71) | 2.16 (0.54,8.71) | 2.01 (0.59,6.85) | 1.65 (0.63,4.35) | 1 | 0.70 (0.19,2.54) | 0.65 (0.17,2.50) | 0.60 (0.26,1.38) |
| imatinib | 7.77 (0.92,65.80) | 3.11 (0.70,13.85) | 2.88 (0.75,11.03) | 2.37 (0.78,7.20) | 1.44 (0.39,5.24) | 1 | 0.94 (0.22,3.98) | 0.86 (0.32,2.33) |
| Pazopanib | 8.31 (0.95,72.47) | 3.32 (0.71,15.45) | 3.08 (0.77,12.36) | 2.54 (0.79,8.15) | 1.54 (0.40,5.88) | 1.07 (0.25,4.55) | 1 | 0.92 (0.32,2.65) |
| Placebo | 9.00 (1.36,59.68) | 3.60 (1.18,11.00) | 3.34 (1.35,8.24) | 2.75 (1.67,4.52) | 1.66 (0.72,3.82) | 1.16 (0.43,3.12) | 1.08 (0.38,3.11) | 1 |

Abbreviations: OR, odds ratio; CI, confidence interval; SCURA, surface under the cumulative ranking curve. Yellow indicated a relative treatment benefit, blue indicated a relative treatment harm, pink indicated no significant.

| Table S6. Matrix of each pairwise comparison of all the agents on 8-month overall survival rate (shown as OR and 95% CI). | | | | | | | | |
| --- | --- | --- | --- | --- | --- | --- | --- | --- |
|  | Masitinib | Pimitespib | Ripretinib | Sunitinib | Imatinib | Regorafenib | Pazopanib | Placebo |
| SCURA (%) | 85.7 | 79.3 | 75.3 | 55.1 | 35.7 | 27.4 | 24.8 | 16.8 |
| Masitinib | 1 | 0.63 (0.06,6.89) | 0.54 (0.05,5.43) | 0.31 (0.04,2.10) | 0.20 (0.02,2.13) | 0.17 (0.02,1.64) | 0.15 (0.01,1.66) | 0.14 (0.02,1.09) |
| Pimitespib | 1.58 (0.15,17.29) | 1 | 0.85 (0.16,4.50) | 0.48 (0.12,1.99) | 0.32 (0.06,1.79) | 0.27 (0.05,1.34) | 0.24 (0.04,1.41) | 0.23 (0.06,0.79) |
| Ripretinib | 1.86 (0.18,18.79) | 1.17 (0.22,6.19) | 1 | 0.57 (0.16,2.04) | 0.38 (0.08,1.88) | 0.31 (0.07,1.40) | 0.29 (0.06,1.49) | 0.27 (0.09,0.79) |
| Sunitinib | 3.27 (0.48,22.51) | 2.07 (0.50,8.49) | 1.76 (0.49,6.31) | 1 | 0.66 (0.17,2.55) | 0.55 (0.16,1.85) | 0.51 (0.13,2.03) | 0.47 (0.24,0.90) |
| Imatinib | 4.93 (0.47,51.75) | 3.11 (0.56,17.31) | 2.65 (0.53,13.22) | 1.51 (0.39,5.78) | 1 | 0.83 (0.17,3.93) | 0.76 (0.14,4.16) | 0.70 (0.22,2.28) |
| Regorafenib | 5.95 (0.61,58.14) | 3.76 (0.75,18.91) | 3.20 (0.71,14.33) | 1.82 (0.54,6.13) | 1.21 (0.25,5.73) | 1 | 0.92 (0.19,4.54) | 0.85 (0.31,2.36) |
| Pazopanib | 6.47 (0.60,69.64) | 4.08 (0.71,23.51) | 3.48 (0.67,18.00) | 1.98 (0.49,7.93) | 1.31 (0.24,7.15) | 1.09 (0.22,5.36) | 1 | 0.92 (0.27,3.14) |
| Placebo | 7.01 (0.91,53.71) | 4.42 (1.27,15.46) | 3.77 (1.26,11.28) | 2.14 (1.11,4.13) | 1.42 (0.44,4.60) | 1.18 (0.42,3.27) | 1.08 (0.32,3.69) | 1 |

Abbreviations: OR, odds ratio; CI, confidence interval; SCURA, surface under the cumulative ranking curve. Yellow indicated a relative treatment benefit, blue indicated a relative treatment harm, pink indicated no significant.

| Table S7. Matrix of each pairwise comparison of all the agents on 9-month overall survival rate (shown as OR and 95% CI). | | | | | | | | |
| --- | --- | --- | --- | --- | --- | --- | --- | --- |
|  | Masitinib | Ripretinib | Sunitinib | Pimitespib | Pazopanib | Regorafenib | Placebo | Imatinib |
| SCURA (%) | 89.3 | 77.7 | 71.7 | 67.8 | 31.2 | 21.9 | 20.4 | 20.0 |
| Masitinib | 1 | 0.56 (0.09,3.31) | 0.48 (0.10,2.32) | 0.45 (0.07,2.91) | 0.21 (0.03,1.35) | 0.18 (0.03,1.02) | 0.18 (0.04,0.89) | 0.17 (0.03,1.06) |
| Ripretinib | 1.79 (0.30,10.61) | 1 | 0.86 (0.37,1.97) | 0.81 (0.25,2.69) | 0.38 (0.12,1.25) | 0.32 (0.12,0.87) | 0.32 (0.15,0.68) | 0.30 (0.09,0.97) |
| Sunitinib | 2.08 (0.43,10.04) | 1.16 (0.51,2.67) | 1 | 0.94 (0.35,2.54) | 0.44 (0.17,1.17) | 0.37 (0.18,0.78) | 0.37 (0.26,0.52) | 0.35 (0.14,0.91) |
| Pimitespib | 2.20 (0.34,14.14) | 1.23 (0.37,4.08) | 1.06 (0.39,2.85) | 1 | 0.47 (0.13,1.72) | 0.39 (0.13,1.23) | 0.39 (0.15,0.99) | 0.37 (0.10,1.35) |
| Pazopanib | 4.72 (0.74,30.10) | 2.64 (0.80,8.66) | 2.27 (0.85,6.03) | 2.14 (0.58,7.89) | 1 | 0.84 (0.27,2.60) | 0.83 (0.33,2.09) | 0.80 (0.22,2.86) |
| Regorafenib | 5.59 (0.98,31.79) | 3.12 (1.15,8.49) | 2.69 (1.29,5.61) | 2.54 (0.82,7.89) | 1.19 (0.38,3.65) | 1 | 0.99 (0.52,1.90) | 0.95 (0.32,2.84) |
| Placebo | 5.65 (1.13,28.29) | 3.16 (1.48,6.73) | 2.72 (1.93,3.82) | 2.56 (1.01,6.49) | 1.20 (0.48,3.00) | 1.01 (0.53,1.94) | 1 | 0.96 (0.40,2.32) |
| Imatinib | 5.90 (0.94,37.00) | 3.30 (1.03,10.54) | 2.83 (1.10,7.30) | 2.68 (0.74,9.63) | 1.25 (0.35,4.47) | 1.05 (0.35,3.16) | 1.04 (0.43,2.52) | 1 |

Abbreviations: OR, odds ratio; CI, confidence interval; SCURA, surface under the cumulative ranking curve. Yellow indicated a relative treatment benefit, blue indicated a relative treatment harm, pink indicated no significant.

| Table S8. Matrix of each pairwise comparison of all the agents on 10-month overall survival rate (shown as OR and 95% CI). | | | | | | | | |
| --- | --- | --- | --- | --- | --- | --- | --- | --- |
|  | Masitinib | Ripretinib | Sunitinib | Pimitespib | Pazopanib | Imatinib | Regorafenib | Placebo |
| SCURA (%) | 94.8 | 82.6 | 68.5 | 59.6 | 29.6 | 24.7 | 21.0 | 19.2 |
| Masitinib | 1 | 0.50 (0.10,2.54) | 0.34 (0.09,1.38) | 0.29 (0.05,1.58) | 0.16 (0.03,0.84) | 0.14 (0.03,0.77) | 0.13 (0.03,0.64) | 0.13 (0.03,0.55) |
| Ripretinib | 1.99 (0.39,10.10) | 1 | 0.68 (0.30,1.57) | 0.58 (0.17,1.90) | 0.31 (0.10,1.00) | 0.28 (0.09,0.92) | 0.27 (0.10,0.72) | 0.26 (0.12,0.56) |
| Sunitinib | 2.92 (0.73,11.74) | 1.46 (0.64,3.37) | 1 | 0.84 (0.32,2.24) | 0.45 (0.18,1.18) | 0.41 (0.16,1.08) | 0.39 (0.19,0.81) | 0.38 (0.28,0.54) |
| Pimitespib | 3.47 (0.63,18.98) | 1.74 (0.53,5.75) | 1.19 (0.45,3.16) | 1 | 0.54 (0.15,1.94) | 0.49 (0.13,1.78) | 0.46 (0.15,1.42) | 0.46 (0.18,1.14) |
| Pazopanib | 6.44 (1.19,34.74) | 3.23 (1.00,10.46) | 2.20 (0.85,5.71) | 1.86 (0.52,6.68) | 1 | 0.91 (0.25,3.24) | 0.86 (0.28,2.58) | 0.85 (0.35,2.07) |
| Imatinib | 7.09 (1.30,38.62) | 3.56 (1.08,11.67) | 2.43 (0.92,6.39) | 2.05 (0.56,7.45) | 1.10 (0.31,3.94) | 1 | 0.94 (0.31,2.89) | 0.93 (0.38,2.32) |
| Regorafenib | 7.52 (1.56,36.22) | 3.77 (1.38,10.31) | 2.58 (1.24,5.36) | 2.17 (0.70,6.70) | 1.17 (0.39,3.53) | 1.06 (0.35,3.24) | 1 | 0.99 (0.52,1.90) |
| Placebo | 7.60 (1.82,31.78) | 3.81 (1.77,8.20) | 2.60 (1.87,3.63) | 2.19 (0.87,5.50) | 1.18 (0.48,2.88) | 1.07 (0.43,2.66) | 1.01 (0.53,1.94) | 1 |

Abbreviations: OR, odds ratio; CI, confidence interval; SCURA, surface under the cumulative ranking curve. Yellow indicated a relative treatment benefit, blue indicated a relative treatment harm, pink indicated no significant.

| Table S9. Matrix of each pairwise comparison of all the agents on 11-month overall survival rate (shown as OR and 95% CI). | | | | | | | | |
| --- | --- | --- | --- | --- | --- | --- | --- | --- |
|  | Masitinib | Pimitespib | Ripretinib | Sunitinib | Pazopanib | Imatinib | Regorafenib | Placebo |
| SCURA (%) | 88.1 | 75.4 | 68.2 | 54.7 | 36.8 | 29.1 | 26.3 | 21,4 |
| Masitinib | 1 | 0.53 (0.05,6.06) | 0.42 (0.04,4.41) | 0.28 (0.05,1.63) | 0.19 (0.02,2.07) | 0.15 (0.01,1.71) | 0.15 (0.01,1.48) | 0.14 (0.02,1.01) |
| Pimitespib | 1.87 (0.17,21.25) | 1 | 0.78 (0.11,5.58) | 0.53 (0.10,2.81) | 0.35 (0.05,2.65) | 0.29 (0.04,2.19) | 0.27 (0.04,1.87) | 0.27 (0.06,1.15) |
| Ripretinib | 2.39 (0.23,25.23) | 1.28 (0.18,9.11) | 1 | 0.67 (0.14,3.23) | 0.45 (0.06,3.09) | 0.37 (0.05,2.56) | 0.35 (0.06,2.18) | 0.34 (0.09,1.29) |
| Sunitinib | 3.56 (0.61,20.64) | 1.90 (0.36,10.13) | 1.49 (0.31,7.12) | 1 | 0.67 (0.13,3.42) | 0.55 (0.11,2.84) | 0.52 (0.11,2.36) | 0.51 (0.22,1.18) |
| Pazopanib | 5.34 (0.48,59.03) | 2.85 (0.38,21.51) | 2.23 (0.32,15.42) | 1.50 (0.29,7.72) | 1 | 0.82 (0.11,6.06) | 0.77 (0.12,5.16) | 0.77 (0.19,3.13) |
| Imatinib | 6.50 (0.58,72.40) | 3.47 (0.46,26.42) | 2.72 (0.39,18.94) | 1.83 (0.35,9.51) | 1.22 (0.17,8.98) | 1 | 0.94 (0.14,6.34) | 0.93 (0.23,3.86) |
| Regorafenib | 6.89 (0.67,70.53) | 3.68 (0.54,25.32) | 2.88 (0.46,18.06) | 1.94 (0.42,8.88) | 1.29 (0.19,8.59) | 1.06 (0.16,7.12) | 1 | 0.99 (0.28,3.52) |
| Placebo | 6.97 (0.99,48.87) | 3.72 (0.87,15.87) | 2.91 (0.77,10.95) | 1.96 (0.85,4.53) | 1.30 (0.32,5.33) | 1.07 (0.26,4.43) | 1.01 (0.28,3.60) | 1 |

Abbreviations: OR, odds ratio; CI, confidence interval; SCURA, surface under the cumulative ranking curve. Yellow indicated a relative treatment benefit, blue indicated a relative treatment harm, pink indicated no significant.


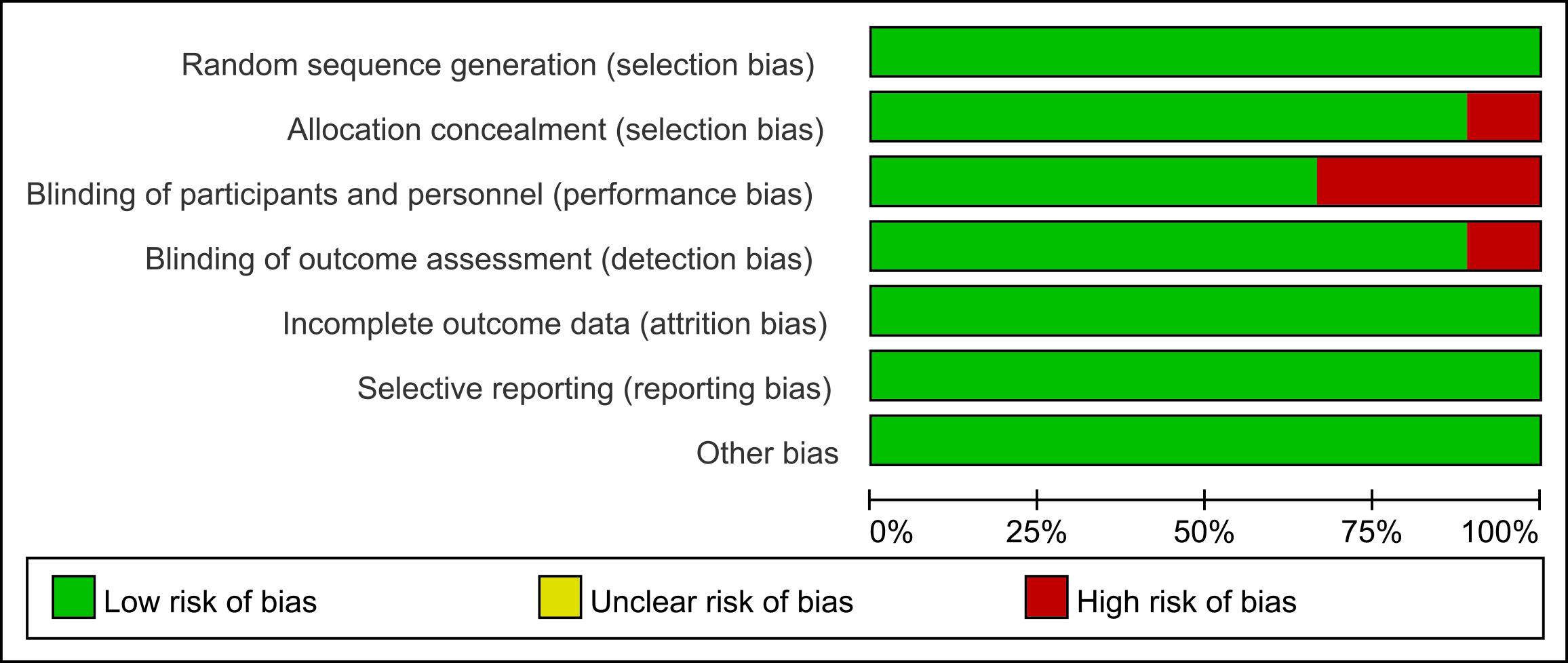


Figure S1. Risk of bias graph.


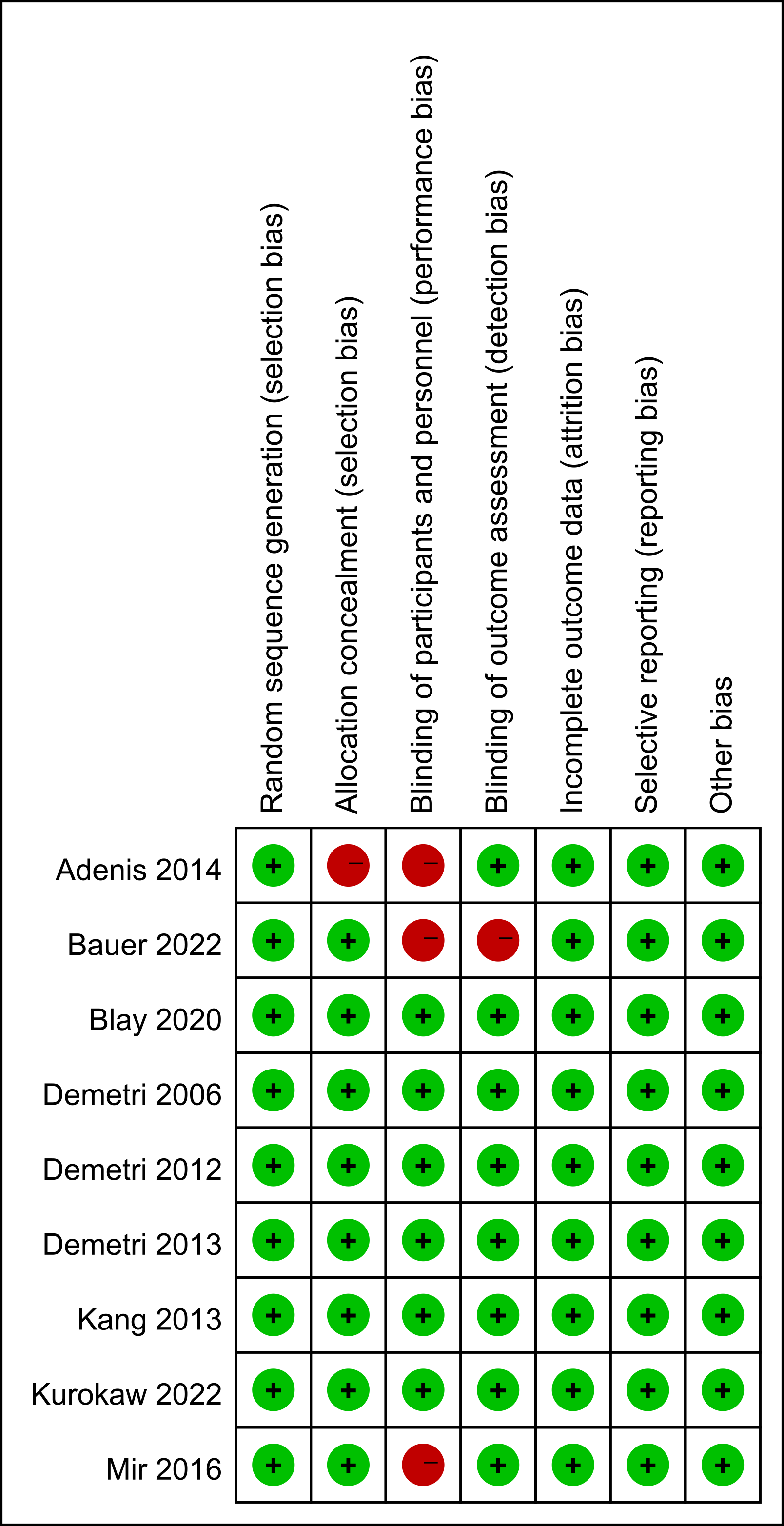


Figure S2. Risk of bias summary; “+” (green) and “-” (red) represent low and high risk of bias, respectively.


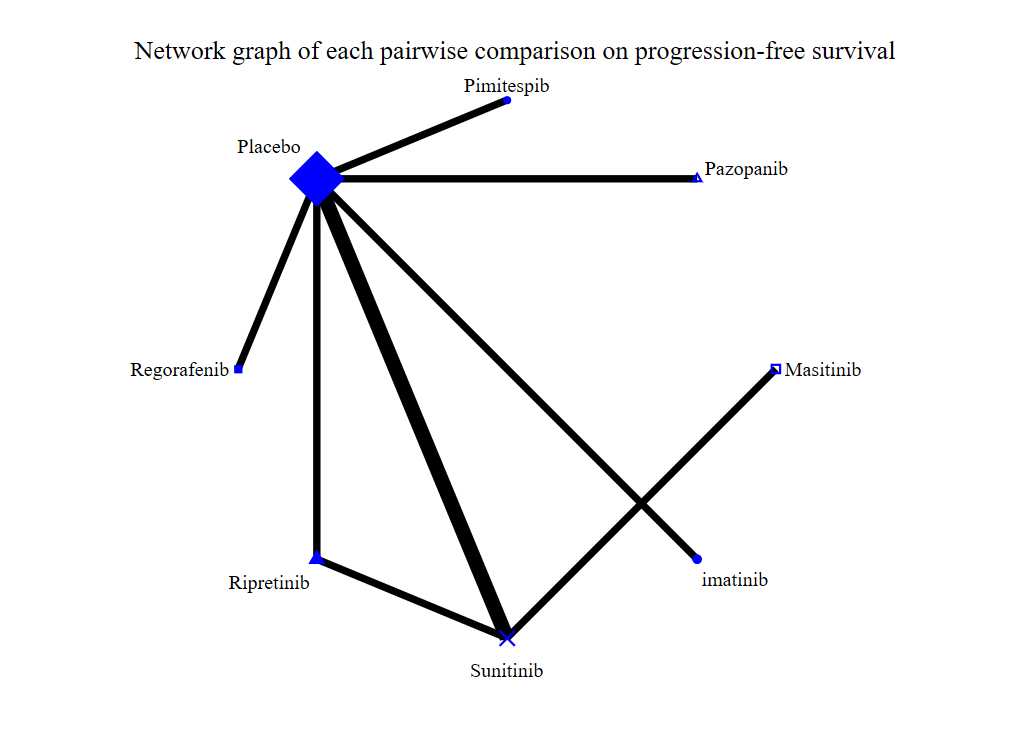


Figure S3. Network graph of each pairwise comparison on progression-free survival.


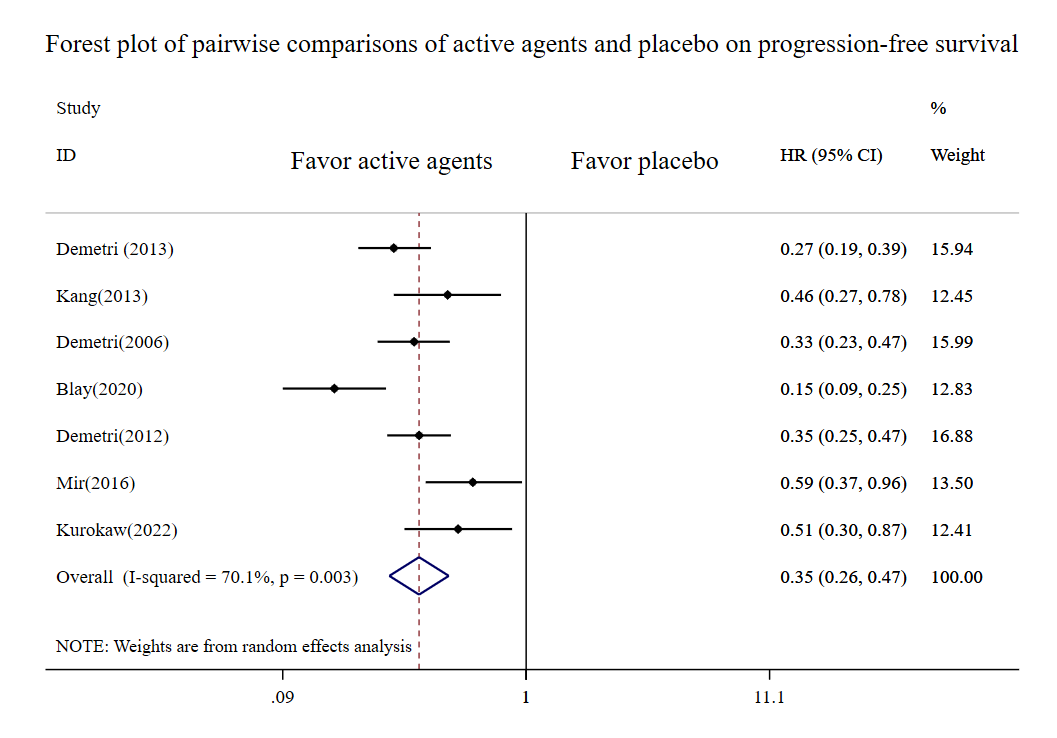


Figure S4. Forest plot of pairwise comparisons of active agents and placebo on progression-free survival.


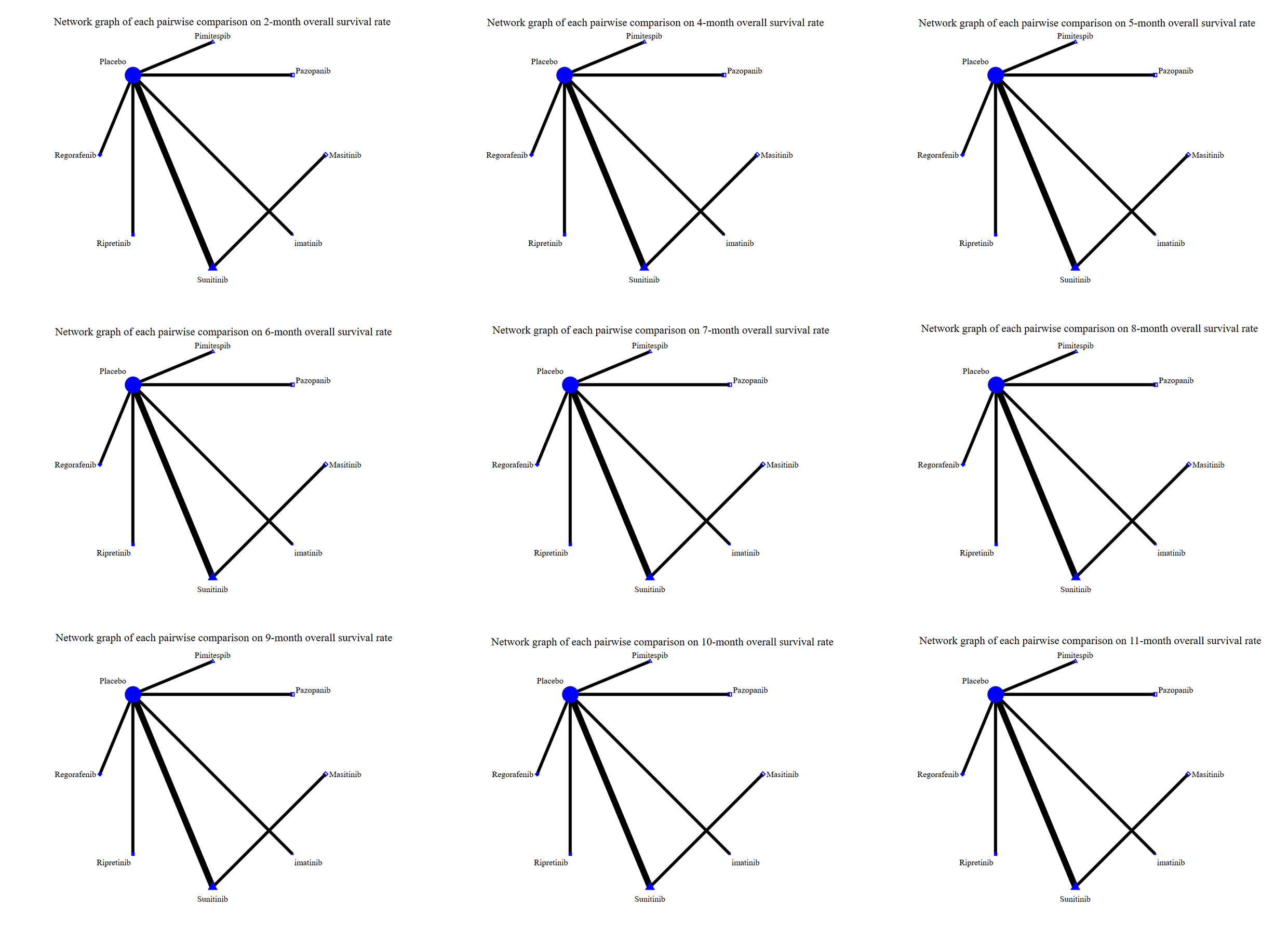


Figure S5. Network graph of each pairwise comparison on overall survival rate at certain time points.


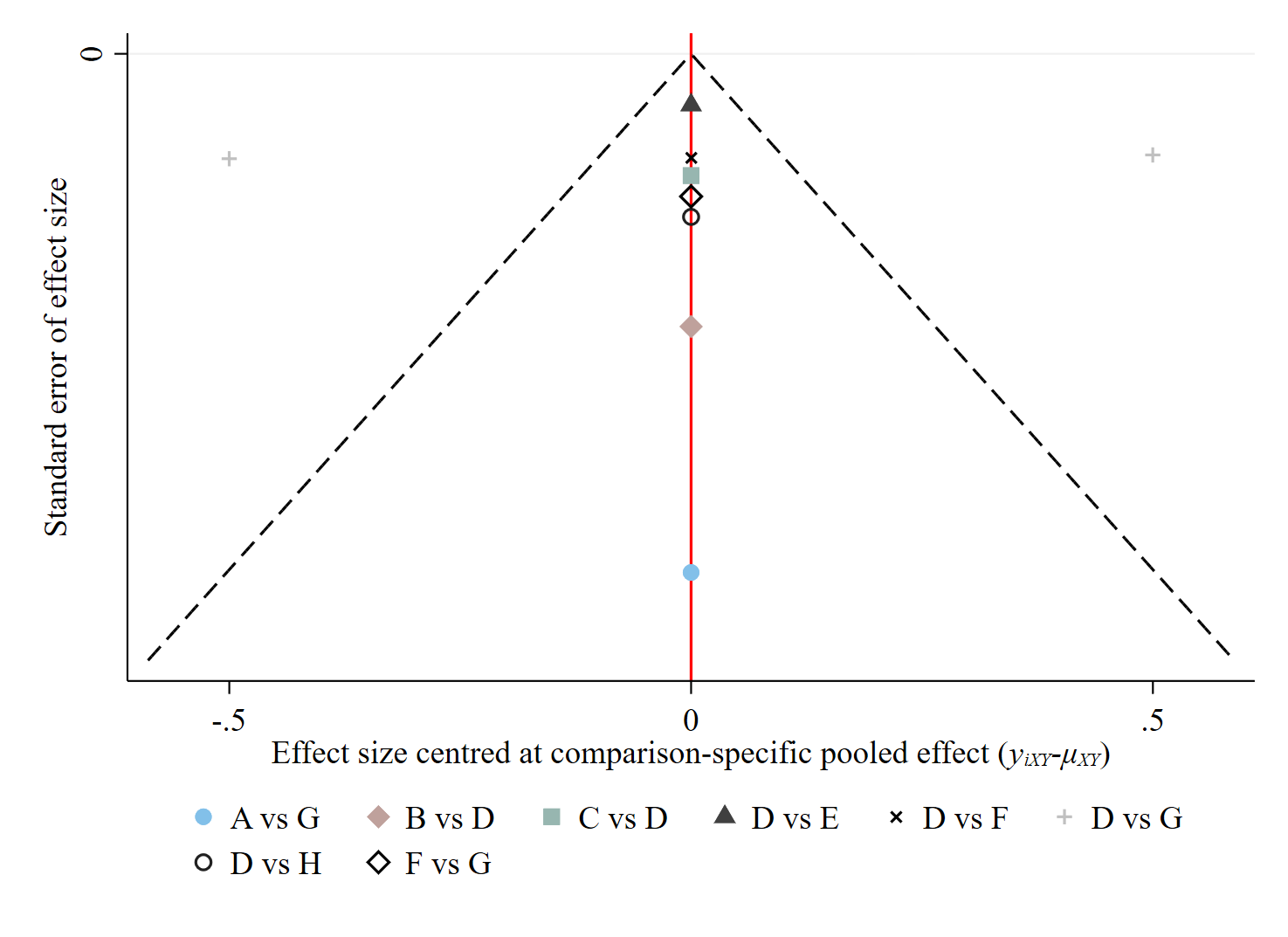


Figure S6. Network funnel plot of pairwise comparisons on progression-free survival; A, Masitinib; B, Pazopanib; C, Pimitespib; D, Placebo; E, Regorafenib; F, Ripretinib; G, Sunitinib; H, Imatinib.


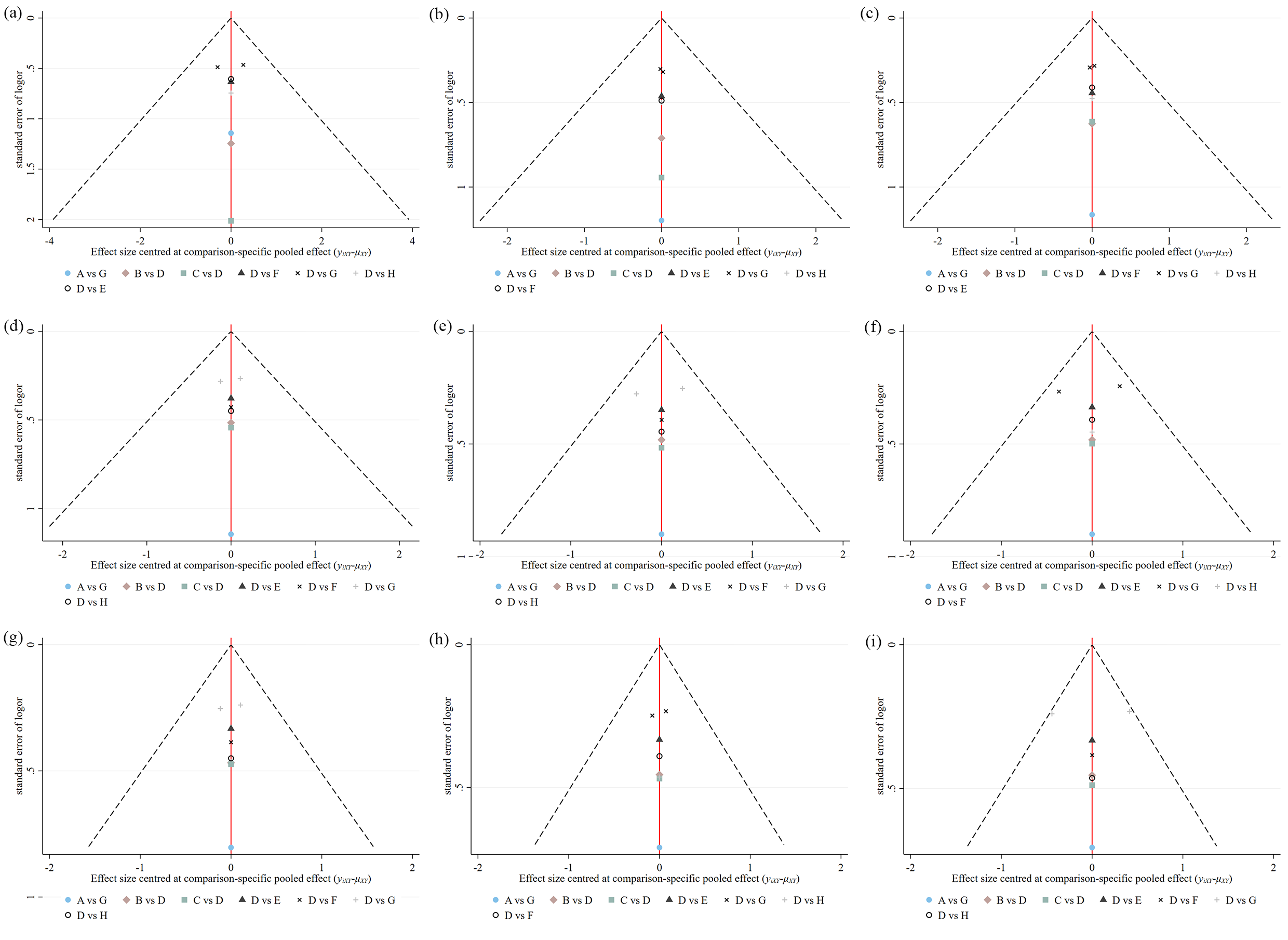


Figure S7. Network funnel plot of pairwise comparisons on overall survival rate at certain time points; (a) 2-month, (b) 4-month, (c) 5-month, (d) 6-month, (e) 7-month, (f) 8-month, (g) 9-month, (h) 10-month, (i) 11-month; A, Masitinib; B, Pazopanib; C, Pimitespib; D, Placebo; E, Regorafenib; F, Ripretinib; G, Sunitinib; H, Imatinib.
